# Intermetallic-anchored epidermal EGaIn patch with analog constriction gates for cardiorespiratory monitoring

**DOI:** 10.1126/sciadv.aee5907

**Published:** 2026-06-26

**Authors:** Yue Li, Yu-chun Lin, Anwei He, Xuejie Wang, Xu Wang, Johnson Q. Cui, Hnin Yin Yin Nyein

**Affiliations:** Department of Chemical and Biological Engineering, The Hong Kong University of Science and Technology, Clear Water Bay, Hong Kong SAR 000000, China.

## Abstract

Chronic respiratory diseases lack accessible tools for continuous, at-home monitoring, as wearable strain sensors struggle to combine accuracy, linearity, and stability. Here, we present HELP (heteromodal epidermal liquid-metal patch), a materials-and-structure co-designed wearable to overcome these limitations. Indium-driven alloying of silver nanowires anchors liquid metal to eliminate drift, while graded hemispherical bulges acting as progressive analog strain gates yield *R*^2^ = 0.998 linearity, <0.4% hysteresis, detection to 0.01% strain, and durability over 500,000 cycles via simple stencil brushing. Its dual channel allows simultaneous strain-insensitive electrocardiogram and ultrasensitive respiratory sensing. In pilot studies, HELP captured thoracoabdominal effort, heart rate, posture, and oxygenation, enabling obstructive sleep apnea detection with high polysomnography agreement, objective tracking of bronchodilator response in asthma, and unmasking of nocturnal hypoxemia hidden by wakeful compensation in chronic obstructive pulmonary disease. By integrating respiratory mechanics and autonomic phenotyping, this self-applied epidermal device establishes a promising platform for future decentralized cardiorespiratory monitoring.

## Teaser

An on-skin patch with engineered liquid-metal circuits tracks breathing and heart activity, uncovering hidden respiratory risks.

## INTRODUCTION

Chronic respiratory diseases (CRDs), including asthma, chronic obstructive pulmonary disease (COPD), and obstructive sleep apnea (OSA), are among the leading causes of morbidity, yet their management continues to rely on diagnostic tools that are largely restricted to clinical environments (*1*–*3*). Although continuous, objective monitoring can reduce exacerbations, hospitalizations, and nocturnal oxygen desaturations, gold standards for lung function and sleep-disordered breathing, such as spirometry and polysomnography (PSG), require specialized facilities, trained personnel, and procedures, making them impractical for children, the elderly, frail individuals, and routine home use (*4*–*7*). As a result, day-to-day disease progression and critical nighttime events often go undetected until the disease was severe. These limitations highlight the urgent need of a comfortable, accurate, at-home cardiorespiratory monitoring solution (*8*, *9*).

Wearable sensors represent a promising avenue for continuous decentralized respiratory monitoring, yet achieving clinical-grade data acquisition in a soft, unobtrusive form factor remains challenging (*10*, *11*). Techniques like respiratory inductive plethysmography can be susceptible to motion artifacts and discomfort (*12*, *13*), while nasal- or mask-based sensors introduce added airway resistance or obtrusiveness that limits long-term use (*14*–*17*). Epidermal resistive strain sensors have thus emerged as an alternative, but their translation to precise volumetric respiratory assessments requires overcoming inherent performance trade-offs (*7*, *18*). Accurately assessing CRDs necessitates a sensing modality capable of capturing a wide dynamic range of strain, from deep breaths (>30% strain) to subtle respiratory efforts (<0.1% strain), with high signal linearity and minimal hysteresis to support accurate tidal-volume estimation (*11*, *19*). Most existing wearable systems are primarily limited to monitoring the respiratory rate, farther than the volumetric metrics essential for a comprehensive diagnostic assessment (*11*).

This performance gap is rooted in fundamental material-level limitations (*20*). Highly sensitive nanomaterials-based strain sensors often rely on microcrack formation or the disruption of percolated nanomaterial networks, which achieve high gauge factors but also introduce pronounced nonlinearity and substantial hysteresis (*21*–*23*). These effects arise from the intrinsic irreversibility of nanoscale conductive pathways, causing nonmonotonic sensor responses that undermine accurate tracking of dynamic signals such as respiration (*22*, *23*). Liquid metals (LMs) offer a fundamentally different approach. Unlike nanomaterials-based sensors that trade stability for sensitivity, LMs have near-zero internal friction and fluidic deformability, enabling virtually fatigue-free, low-hysteresis operation alongside high sensitivity to subtle strains when integrated with appropriate structural designs. However, their practical deployment is tempered by a critical barrier: Poor intrinsic adhesion to common elastomers leads to mechanical decoupling at the LM-substrate interface (*24*, *25*). This interfacial incompatibility allows unconstrained LM motion within microchannels, resulting in signal lag and baseline drift that undermine measurement accuracy (*26*, *27*). Although current confinement strategies, such as ultrathin sealed channels or laser-sintered activation, can mitigate this issue, they often rely on complex and nonscalable fabrication (*28*–*30*). Therefore, realizing a stable, high-performance respiratory sensor necessitates not only the advantageous properties of LMs but also a scalable and robust interfacial strategy that prevents decoupling and ensures a reliable mechanical-electrical coupling (*10*, *31*).

**Fig. 1.**
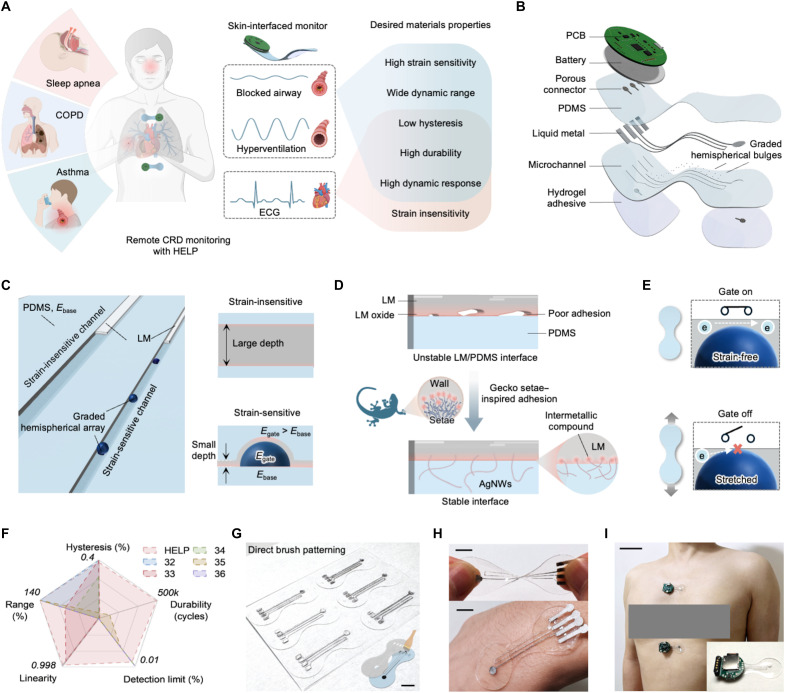
Overview of the HELP for daily monitoring of CRDs. (**A**) Schematic illustration of the HELP system, depicting the acquisition of key physiological signals and the desired material properties. Created in BioRender. Nyein, H. (2026), https://biorender.com/sneexx9. (**B**) Exploded view of the HELP architecture. (**C**) Schematic of the strain-insensitive liquid metal (LM) microchannel for ECG acquisition and the strain-sensitive LM microchannel regulated by gradient hemispheres for respiratory monitoring. (**D**) Bioinspired interfacial modification with embedded AgNW enables strain-matched deformation and durable LM-elastomer adhesion. (**E**) Mechanism of resistance modulation under strain: Progressive closure of hemispherical gates increases channel resistance. (**F**) Radar chart comparing the performance of HELP with state-of-the-art wearable strain sensors (*32*–*36*). (**G**) Image of the stencil-printed LM sensor arrays. Scale bars, 1 cm. (**H**) Images of the sensing patch integrated with porous flexible I/O connectors. Scale bars, 1 cm. (**I**) Photograph of the HELP device placed on the chest and abdomen for multimodal physiological monitoring. Scale bar, 5 cm.

To address these challenges, we introduce HELP (heteromodal-performance epidermal liquid-metal patch), a soft multimodal cardiorespiratory wearable that overcomes the fundamental performance trade-offs in respiration monitoring through an integrated materials-and-structure design (Fig. 1, A and B). A dual-channel LM architecture simultaneously delivers strain-insensitive traces for stable electrocardiogram (ECG) and a U-shaped microchannel optimized for full-range respiratory monitoring (Fig. 1C). Silver nanowire (AgNW) anchors chemically and mechanically couple the LM to polydimethylsiloxane (PDMS), eliminating interfacial decoupling and long-term signal drift (Fig. 1D). Unlike prior approaches that blend metal fillers into LM to modify rheology for printing—often relying on specialized equipment—our strategy uses AgNWs as discrete interfacial anchors embedded in the substrate, enabling a simple, scalable stencil-brushing process without sintering or specialized tools.

Building on this robust interfacial anchoring, the respiratory channel incorporates graded hemispherical bulges that act as progressive analog strain gates (Fig. 1E), enabling detection of subtle breaths (<0.1% strain) while preserving exceptional linearity (*R*^2^ = 0.998) and ultralow hysteresis (<0.4%) across strains exceeding >30%. This unique combination of performance metrics surpasses previously reported wearable strain sensors (Fig. 1F and table S1) (*32*–*36*) and is achieved through a simple stencil-brushing fabrication process (Fig. 1G).

Integrated with porous flexible I/O (input/output) connectors (Fig. 1H) and a miniature wireless printed circuit board (PCB) with an inertial measurement unit, HELP enables <1-min self-application and continuous monitoring of respiration, ECG, and physical activity in daily life (Fig. 1I). In human studies, HELP detected sleep apnea events with accuracy comparable to PSG, captured bronchodilator-induced breathing improvements in asthma, and revealed nocturnal hypoxemia together with the loss of wakeful compensatory breathing in a COPD participant, a capability enabled by intrinsic signal fidelity rather than algorithmic postprocessing, demonstrating its potential for decentralized, longitudinal cardiorespiratory phenotyping.

## RESULTS

### Materials design and characterization for the multimodal sensing patch

**Fig. 2.**
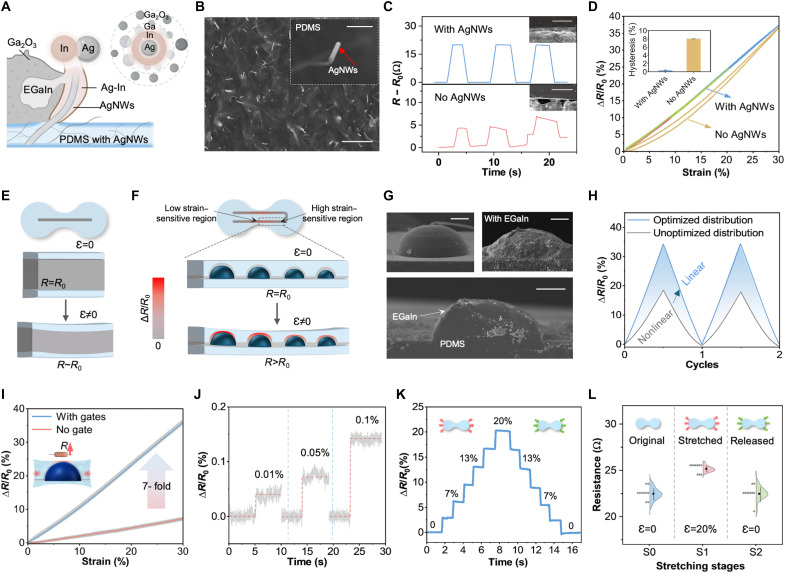
Characterization of interfacial anchoring and gradient-gated microchannel design of the HELP sensor. (**A**) Schematic of the AgNW-mediated LM anchoring mechanism. (**B**) SEM image showing AgNWs partially embedded in PDMS. Scale bars, 10 μm (main image) and 1 μm (inset). (**C**) Electrical response and corresponding SEM micrographs of the LM-PDMS interface with and without AgNWs upon repeated stretching. Scale bars, 500 μm. (**D**) Hysteresis loops of LM channels with and without AgNWs. The AgNW-anchored channel is shown across 10, 20, and 30% maximum strain. Inset: Comparison of hysteresis values for both channel types. Error bars represent the standard deviation across sensors (*n* = 3). (**E**) Schematic of the strain-insensitive LM channel for ECG sensing. (**F**) Schematic of the strain-sensitive LM channel incorporating graded hemispherical constriction gates for respiratory monitoring. (**G**) SEM images of the hemispherical gates with and without EGaIn coating. Scale bars, 50 μm. (**H**) Resistance-applied strain for the LM microchannel with an unoptimized (nongraded) gate distribution against the optimized (spatially graded) design. (**I**) Sensitivity comparison between gradient-gated and smooth (ungated) channels. Gray error bars represent the SD of the mean from three sensors. (**J**) Subtle strain detection performance from 0.01 to 0.1% strain. (**K**) Dynamic sensing response of the U-shape channel under cyclic strain. (**L**) Reproducibility of sensors demonstrated by resistance changes after 20 cycles of 20% strain. Error bars represent the SD of the mean from 10 sensors.

Achieving high-fidelity cardiorespiratory signals requires solving the fundamental challenge of unstable LM containment within stretchable elastomers. Conventional LM-based microchannels suffer from interfacial decoupling because eutectic gallium-indium (EGaIn) poorly wets PDMS, leading to signal drift and pronounced hysteresis under strain. To address this limitation, we developed a bioinspired anchoring strategy that couples chemical bonding with mechanical interlocking at the LM-PDMS interface. This is achieved by predepositing a network of AgNWs on the PDMS surface, mimicking the hierarchical fibrillar structure of gecko setae, and formulating the LM into a stable paste by adding trace metal microparticles, which fracture its bulk oxide into dispersible flakes. During a simple, scalable stencil-brushing process, repeated shear ruptures the oxide shells of the paste, exposing fresh LM cores. These cores rapidly alloy with the AgNWs through indium, disrupt further oxide formation, and enable spontaneous wetting of and adhesion to PDMS (Fig. 2A) (*37*–*39*). Simultaneously, the high aspect ratio of AgNWs allows deep embedding and interlocking within the PDMS matrix, forming a robust, multipoint anchoring architecture (Fig. 2B) that prevents decohesion (figs. S1 and S2).

To evaluate the effect of AgNW density on LM-PDMS interfacial reliability, we measured resistance stability over 40 cycles at 2% strain to assess initial small-strain stability and over 1000 cycles at 20% strain to assess long-term durability across AgNW surface densities ranging from 0 to 89.76 wt % (energy dispersive x-ray spectroscopy surface coverage). Below ~4.7 wt %, insufficient AgNWs led to poor LM anchoring, resulting in large resistance fluctuations. Above ~45 wt %, excessive Ag-In intermetallic formation caused progressive resistance degradation under cycling. An intermediate density range enabled stable performance, demonstrating that controlled AgNW density is critical for balancing mechanical anchoring and metallurgical compatibility (fig. S3). At the optimized AgNW density (4.7 wt %, as used in subsequent study), the alloying reaction is confined to the nanoscale tips of the semiembedded AgNWs, creating sparse, discrete anchoring points. This preserves the fluidic properties of the bulk EGaIn and prevents cracking or mechanical failure even after extensive cycling. The resulting continuous LM thin films deform synchronously with the substrate, substantially suppressing device hysteresis from 8 to 0.4% at 30% strain under cyclic loading (Fig. 2, C and D). This simple stencil-brushing process, enabled by the AgNW anchoring strategy, eliminates the need for sintering or specialized equipment (fig. S4).

This anchored-LM platform enabled a dual-functionality patch comprising a wide, thick channel for strain-insensitive ECG monitoring (Fig. 2E) and a thin U-shaped microchannel for respiratory strain sensing (Fig. 2F). The U-shape is oriented so that its sensing arms align with the primary axis of chest expansion, optimizing the sensor to measure the direct lateral strain. To achieve high sensitivity and linearity, the respiratory channel incorporates graded hemispherical bulges with a higher modulus than the channel walls (Fig. 2G). Under strain, the compliant channel walls deform preferentially, while the rigid bulges progressively constrict the LM’s conductive pathway. Larger bulges close first during mild deformation, enhancing sensitivity to subtle breaths, while smaller bulges engage under greater strains, producing a smooth, highly linear resistance change across the full physiological range (Fig. 2H).

The gate architecture was systematically optimized by varying bulge diameter, spacing, and spatial distribution (note S1 and figs. S5 to S7). To establish a theoretical foundation, we developed an analytical model and Hertz contact-based numerical simulations. The analytical model shows that sensor linearity is governed by radius dispersion and sensitivity scales with gate density—relationships validated by numerical simulation, which confirms that graded designs outperform uniform designs and an optimal balance exists (note S1 and fig. S8). Designs with uniform bulge size were suboptimal, as they could not accommodate the nonuniform strain field across the chest. The optimal gradient places large bulges in the midregion and smaller ones toward the edges, mirroring the operational strain gradient. During inhalation, the midregion experiences the greatest deformation, causing the large gates to constrict first and providing high sensitivity to weak breaths. As strain increases, the smaller gates engage sequentially, linearizing the response and preventing signal saturation during deep inhalation. This configuration produces smooth, progressive constriction of the LM pathway and achieves exceptional linearity (*R*^2^ = 0.998) over the critical 0 to 30% strain regime, with performance maintained up to 140% strain (*R*^2^ = 0.996) (Fig. 2H and fig. S9). Compared to a smooth-channel control (Fig. 2I), the gradient-gated design provides a sevenfold increase in sensitivity, enabling detection of strains down to 0.01% (Fig. 2J). The sensor also exhibited rapid, stable, and accurate responses to dynamic strain inputs (Fig. 2K) and strong batch-to-batch reproducibility (Fig. 2L). Last, its minimal hysteresis—an intrinsic advantage of this LM-based geometric transduction mechanism—compares favorably with a graphene-based nanocomposite sensor, which shows substantially higher energy dissipation (fig. S10).

### Integrated device performance and validation

**Fig. 3.**
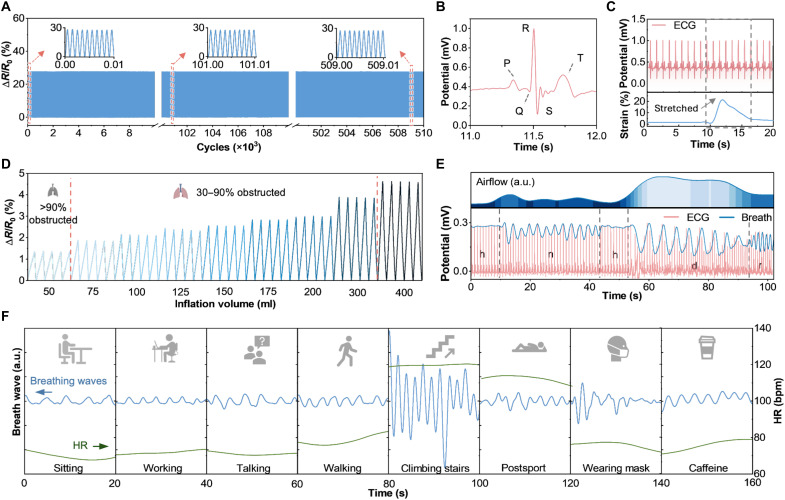
Performance validation and multimodal monitoring capabilities of the HELP system. (**A**) Long-term stability test of the HELP system at 20% strain. (**B**) ECG waveform validated using a commercial electrophysiological simulator (1 mV, 60 bpm), showing distinct P, QRS, and T features. (**C**) Simultaneous ECG and mechanical strain measurement during >20% stretching. The dashed box indicates the period of stretching and recovery. (**D**) Tidal-volume sensitivity of the HELP respiratory channel in a 3-liter lung-simulator balloon, demonstrating reliable detection of volume changes as small as 25 ml. (**E**) Representative respiratory waveforms (inferred airflow volume overlaid) and concurrent ECG during breath holding (h), normal breathing (n), deep inhalation (d), and rapid breathing (r). a.u., arbitrary units. (**F**) Heart rate (HR) and respiratory responses to daily activities. Data were recorded under standardized conditions: during activities (sitting to stair climbing), within 1 min postsport, after 30 to 60 min of mask wearing, and 1 to 2 hours after caffeine intake.

The foundational material platform was subsequently translated into a fully assembled, robust wearable device. A critical final step was the development of a reliable interfacing strategy using porous conductive connectors, which are partially embedded into the LM traces and infiltrated with PDMS to form a monolithic, leak-proof seal (fig. S11 and movie S1). The resulting devices exhibited exceptional durability, showing only 0.07% performance degradation over 500,000 stretching cycles at 20% strain (Fig. 3A), effective suppression of overshoot (note S2 and fig. S12), and stable operation across different replicates and even over 180 days of testing (fig. S13, A and B). The skin interface consists solely of medical-grade adhesive and PDMS, with the LM and AgNWs fully encapsulated; cross-sectional scanning electron microscopy (SEM) after 180 days confirmed no material leakage (fig. S13C), ensuring the safety of the design.

The system’s performance was first validated through benchtop models simulating cardiac and respiratory functions. The ECG subsystem was evaluated using a commercial simulator delivering a stable 1-mV, 60–beats per minute (bpm) test signal, confirming accurate acquisition of standard waveform morphologies with clear P-QRS-T complexes (Fig. 3B). This signal fidelity stems from the dedicated thick-channel LM architecture, which maintains negligible resistance variation even under strain exceeding 20% (Fig. 3C). The quantitative gas-volume response of the sensor was first characterized using a precisely controlled balloon-based lung simulator (fig. S14), which provided a well-defined, repeatable volume-displacement input. This setup allowed us to establish a baseline transfer function between mechanical strain and known air volume over the range of 50 to 400 ml, demonstrating a volume-change resolution of 25 ml and a linear response across simulated tidal volumes (Fig. 3D and fig. S14B). Building on this controlled calibration, we then derived a resistance-to-tidal-volume model from synchronous recordings on human subjects, where HELP signals were correlated with spirometer-measured volumes across the physiological range (fig. S15). Detailed calibration protocols, including the intersubject variability study, are provided in note S3. This two-step approach ensured that the fundamental sensitivity of the sensor was quantified under ideal mechanical coupling, while the human data validated its performance and linearity under realistic, complex thoracic movement. In a combined dynamic test, the device simultaneously maintained a rapid and stable respiratory response across physiological breathing rates (0.1 to 1 Hz) and clean ECG signals across matching stretching frequencies (figs. S16 and S17). This performance, covering the progression from slow breathing to tachypnea, confirms robust, interference-free multimodal acquisition against intrinsic cross-talk.

During on-body testing with structured breathing maneuvers, including breath holding, normal breathing, deep inhalation, and rapid breathing, HELP simultaneously recorded ECG and respiratory signals (Fig. 3E). The respiratory waveforms closely matched ECG-derived respiration patterns, successfully capturing all volitional transitions. Subsequently, HELP was deployed for continuous cardiorespiratory monitoring during diverse daily activities (representative results are shown in Fig. 3F, and additional measurements from different subjects for selected activities are provided in fig. S17). The system successfully discriminated between distinct physiological states, detecting marked increases in breathing and heart rate during exercise and caffeine intake, as well as more subtle variations associated with cognitive load, speech, and mask wearing (*40*). Together, these results establish HELP as a robust platform for multimodal, continuous cardiorespiratory monitoring in real-world conditions.

### Pilot study: At-home screening for sleep apnea

**Fig. 4.**
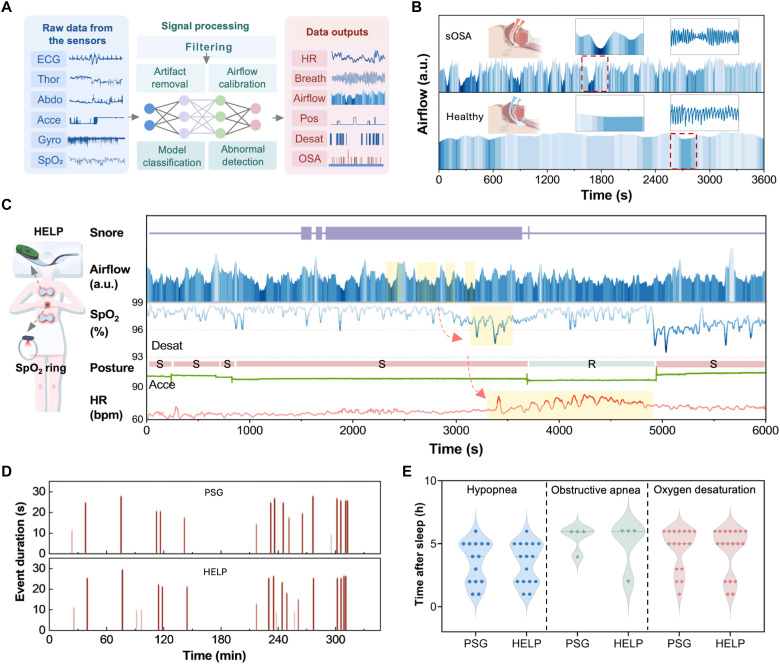
At-home detection and validation of obstructive sleep apnea (OSA) using the HELP system. (**A**) Schematic of the multimodal signal-processing pipeline, including motion-artifact rejection, posture classification, and machine learning–based apnea-hypopnea event detection. Abbreviations: Thor, thoracic movement; abdo, abdominal movement; acce, accelerometer; gyro, gyroscope; pos, posture; desat, oxygen desaturation. (**B**) Representative airflow waveforms inferred from breath wave signals in a suspected OSA (sOSA) participant and a healthy control during sleep. Created in BioRender. H. Y. Y. Nyein (2026), https://biorender.com/sneexx9. (**C**) Representative overnight multimodal signals acquired by the HELP system and an SpO_2_ ring. Posture states: S, supine; R, right lateral. An obstructive apnea sequence with oxygen desaturation and arousal is highlighted in yellow. (**D**) Simultaneous event-by-event comparison of apnea-hypopnea detection between in-laboratory PSG and HELP in the same participant. (**E**) Hourly comparison of apnea/hypopnea and oxygen desaturation events between PSG and HELP across the full night. h, hours.

A major challenge in OSA management is the absence of accessible tools capable of capturing its core pathophysiology: repeated upper-airway collapse followed by the characteristic cascade of respiratory effort without airflow, oxygen desaturation, and autonomic cardiovascular activation (*41*,*42*). Here, we deployed the HELP system that monitors the entire sequence using dual respiratory strain sensors on the chest and abdomen to quantify thoracoabdominal effort, a single-lead ECG to track heart rate and autonomic responses, and a pulse oximeter ring for peripheral oxygen saturation (SpO_2_) measurement (fig. S18). An onboard accelerometer and gyroscope in the HELP system further mitigate motion artifacts, enable robust signal cleaning (Fig. 4A), and identify sleep posture—a known modulator of OSA severity (figs. S19 to S21). This sensor fusion allows HELP to capture the complete cardiorespiratory signature of obstructive events in both laboratory and real-world settings, with the hardware preserving pathological features as discernible raw signals and the machine-learning framework enabling automated, consistent event classification from these high-quality data.

For unsupervised home sleep studies, full-night data were stored on an onboard memory card for subsequent offline analysis (see PCB design; fig. S22). Initial home recordings immediately highlighted clear physiological differences: A participant with high body mass index and habitual snoring exhibited markedly irregular breathing throughout the night, in contrast to a lean individual (Fig. 4B). Detailed inspection of full overnight data of a habitual-snoring participant revealed apnea cycles: During supine sleep, a prolonged obstructive episode (2200 to 3200 s) with audible snoring produced a classic cascade—complete loss of respiratory effort-airflow coupling, a subsequent SpO_2_ drop to the lowest (3100 to 3600 s), and pronounced tachycardia as autonomic arousal restored airway patency (3300 to 4800 s) (Fig. 4C). Identical integrated cardiorespiratory sequences were consistently observed in an additional home-recorded subject (fig. S23), confirming HELP’s ability to capture key physiological signatures outside the traditional sleep laboratory.

To benchmark the performance of the system, we performed simultaneous overnight recordings with gold-standard PSG (fig. S24). Whereas PSG requires >20 wired sensors and a professional setup, HELP was self-applied in <1 min. Respiratory effort, heart-rate variability, body posture, and SpO_2_ data (fig. S25) were fused using a machine-learning framework to detect apnea and hypopnea events (fig. S26). Event timings predicted by HELP showed strong agreement with PSG annotations (Fig. 4D), and the HELP-derived apnea-hypopnea index differed from PSG by only 0.5 events/hour, yielding 89.8% overall agreement and Cohen’s κ of 0.78 (Fig. 4E). This progression from unsupervised home monitoring of complete pathophysiological sequences to preliminary validation against the gold standard shows promise for OSA screening with HELP and suggests its potential as a practical tool for scalable, decentralized sleep-disordered breathing assessment.

### Case studies in asthma and COPD: Revealing compensatory mechanisms and nocturnal decline in chronic airway disease

Patients with asthma and COPD frequently adapt to chronic dyspnea, masking progressive functional decline until acute exacerbations occur (*43*). Therefore, objective, longitudinal respiratory monitoring is essential for detecting subclinical deterioration and enabling preemptive intervention before exacerbations. Leveraging the high sensitivity and low hysteresis of its respiratory strain sensors, we deployed the HELP system to track daily breathing patterns in in-depth case studies of healthy controls and in individuals with asthma or COPD.

**Fig. 5.**
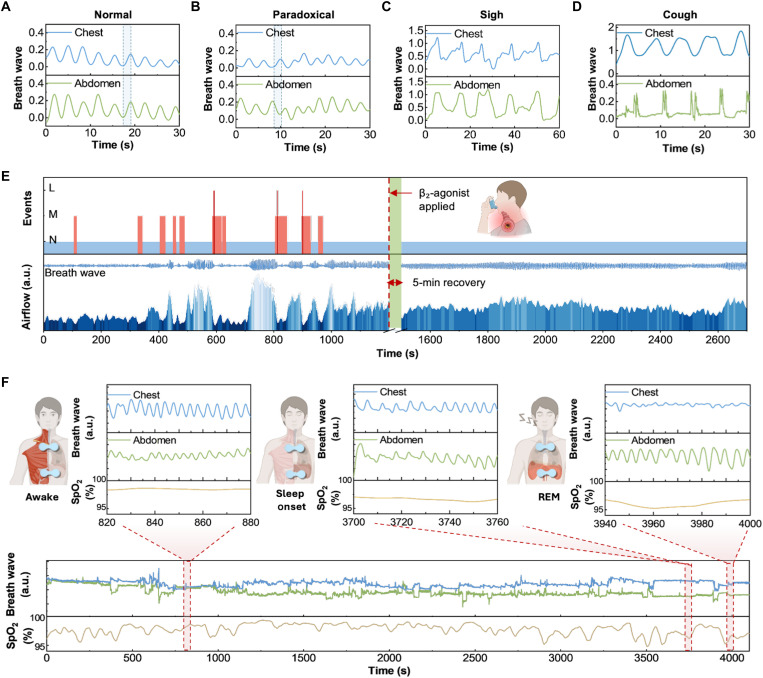
Respiratory signatures of asthma and COPD and acute bronchodilator response captured by the HELP system. (**A**) Normal synchronous thoracoabdominal breathing in a healthy participant. (**B**) Paradoxical thoracoabdominal motion in an asthma participant. Shaded regions indicate periods of asynchrony or phase opposition. (**C**) Frequent sighing pattern observed in a participant with asthma. (**D**) Coughing bursts detected in thoracic and abdominal effort. (**E**) Acute therapeutic response in an asthma participant: prolonged airflow limitation before bronchodilator administration and rapid restoration of normal synchronous breathing after inhalation of a short-acting β_2_-agonist. Labels N, M, and L denote normal, moderately limited, and severely limited airflow, respectively. Created in BioRender. Nyein, H. (2026), https://biorender.com/sneexx9. (**F**) Continuous thoracoabdominal effort and SpO_2_ recording in a participant with moderate-to-severe COPD across wakefulness, sleep onset, and REM sleep. Created in BioRender. Nyein, H. (2026), https://biorender.com/sneexx9.

In healthy individuals, thoracic and abdominal respiratory efforts were highly synchronous, with consistent amplitude and phase coherence (Fig. 5A and fig. S27). In contrast, a participant with asthma exhibited distinct pathological patterns, including paradoxical thoracoabdominal motion [a marker of increased work of breathing and diaphragmatic fatigue (*44*)], episodic hyperventilation, frequent sighing, and coughing bursts (Fig. 5, B to D, and fig. S28). To evaluate the system’s utility for tracking the treatment response, we monitored a participant with asthma overnight before and after bronchodilator use (Fig. 5E). HELP detected a 20-min episode of airflow limitation and hypopnea during sleep. Within 5 min of administering a prescribed short-acting β_2_-agonist, respiratory effort normalized and stable breathing resumed, objectively confirming the intervention’s efficacy.

We next investigated sleep-related respiratory dynamics in a participant with moderate-to-severe COPD, where nocturnal hypoxemia is common yet often undetected (*45*). The ultralow hysteresis and high sensitivity of HELP enabled artifact-free tracking of subtle chest and abdominal movements across the wake-sleep transition. While awake, the participant actively recruited accessory thoracic muscles to maintain SpO_2_ > 98% (Fig. 5F). At sleep onset, the respiratory rate fell from 16 to 13 breaths/min, the expiratory time lengthened markedly [suggesting airflow limitation (*46*)], and abdominal contribution increased to compensate as SpO_2_ declined to ~96%. During apparent rapid eye movement (REM) sleep, thoracic excursions nearly ceased, consistent with physiological muscle atonia, forcing almost exclusive abdominal breathing and a further SpO_2_ drop to ~95%. This unmasked nocturnal hypoxemia that remained fully compensated and undetectable while awake.

These findings highlight a critical yet underrecognized phenomenon: Individuals with COPD may appear stable during waking hours because of voluntary compensatory strategies that collapse during sleep (*47*). This case study illustrates how the high-fidelity signals from HELP, by capturing the full dynamic range from vigorous accessory muscle recruitment to the faint movements of REM-related atonia, can unmask otherwise hidden respiratory decline. Such objective, continuous monitoring provides a window into subclinical deterioration, supports the evaluation of interventions (e.g., supplemental oxygen or ventilatory support), and enables data-driven insights into chronic airway disease progression beyond subjective symptom reports.

## DISCUSSION

A persistent challenge in wearable strain-sensor development is achieving high sensitivity and linearity without sacrificing long-term mechanical or electrical drift. Most nanomaterial-based sensors gain sensitivity at the cost of pronounced hysteresis and irreversible conductive-pathway disruption, whereas conventional liquid-metal channels suffer from interfacial slip and baseline drift (*48*). Through synergistic chemical (AgNW-indium alloying) and mechanical (gecko-inspired nanoanchoring) interfacial engineering, combined with geometrically programmed analog constriction gates, the HELP system simultaneously delivers ultralow strain detection with exceptional linearity, low hysteresis, and high stability. Notably, HELP achieves dual functionality using the same liquid-metal platform: The ECG channel leverages LM’s intrinsic fluidity for strain insensitivity (<10-Ω variation, negligible versus kΩ skin impedance), while the respiratory channel uses engineered geometric constraints (thin film and constricting bulges) to amplify the strain response. This performance profile directly addresses the unmet need for a wearable sensor capable of capturing not just the respiratory rate but accurate tidal volume and pathological breathing patterns—a capability that existing stretchable conductors cannot simultaneously provide.

This convergence of properties has immediate physiological consequences. The combination of ultralow hysteresis and wide dynamic range enables reconstruction of tidal waveforms and subtle thoracoabdominal asynchrony. This capability enables the direct identification of mechanistically informative signatures from raw traces without elaborate postprocessing. These signatures include sustained thoracoabdominal paradox, prolonged expiratory timing, apnea-related tachycardia, rapid reversal of obstruction after bronchodilator inhalation in asthma, and loss of wakeful compensation with nocturnal desaturation in COPD. The platform’s ability to reveal these diverse phenomena reflects intrinsic signal fidelity rather than algorithmic compensation. Constituting a pilot feasibility investigation, the current results—while demonstrating compelling physiological fidelity—are based on a limited cohort and single-night recordings. Larger, longitudinal studies across diverse populations will be essential to establish normative baselines, disease-specific diagnostic thresholds, and long-term sensor reliability. In parallel, fully automated, real-time analysis pipelines for the multimodal data streams remain an important next engineering target.

In summary, by reconciling the historic trade-off between sensitivity and stability in soft strain sensing, HELP provides a wearable interface to lab-grade respiratory mechanics and cardiorespiratory phenotyping. We envision that this hardware foundation can enable a shift from reactive, symptom-based management to proactive, physiology-driven monitoring of chronic respiratory and sleep disorders.

## MATERIALS AND METHODS

### Materials

The PDMS silicone elastomer kit (Sylgard 184) was supplied by the Dow Chemical Co. EGaIn (relative weight ratio, 75.5:24.5) was purchased from Sigma-Aldrich. Spherical nickel powder (1-μm particle size) was procured from Leber Metal Materials Technology Co., Ltd. (Hebei, China). Medical-grade adhesive gel was supplied by Sekisui Kasei Co., Ltd. AgNW solution (nanowires with a 100-nm length and a 50-nm diameter, dispersed in ethanol) was purchased from XFNANO Technology Co., Ltd. (Jiangsu, China).

### Fabrication of the HELP system

A 2-ml ethanol solution of AgNWs (5 mg ml^−1^) was spray coated onto a custom-designed copper mold featuring hemispherical cavities and microchannel patterns (fabricated at the Materials, Design and Manufacturing Facility, The Hong Kong University of Science and Technology). The AgNW density was controlled by two methods, spray-coating to achieve lower Ag content (3.95 to 45.01 wt %) with varying coating layers (1, 2, 3, 5, 10, 15, 20, and 100) and direct brushing to achieve higher Ag content (61.71 to 89.76 wt %), and the resulting Ag content was quantified by energy dispersive x-ray spectroscopy. A layer of PDMS (base-to-curing agent ratio, 5:1) was carefully applied into the cavity regions by drop-casting with a fine syringe needle to form high–Young’s modulus microbulges as cured. Then, a second layer of PDMS (15:1) was cast over the entire mold to form the microchannels and the main body of the patch. PDMS penetrated the AgNW network. After demolding, the AgNWs remained partially embedded on the surface. A 50-μm-thick polyethylene terephthalate stencil mask, fabricated by laser cutting (PLS6. 150D, Universal Laser Systems Inc.), was then aligned and attached to the PDMS substrate. LM paste was prepared by mechanically mixing EGaIn and nickel microparticles at a weight ratio of 3:2 (EGaIn:Ni) using a mortar. The paste was uniformly brushed through the stencil to form the desired circuit pattern on PDMS. The ECG channel measures 400 μm in width and 200 μm in depth. For the respiratory (U-shaped) channel, its width corresponds to the diameter of the hemispherical bulges (200 μm), while its height is determined by the thickness of the brushed LM film (~3 μm). This single-pass thickness (~3 μm) is highly reproducible and optimally balances conductivity with low power consumption for overnight monitoring. After removing the stencil, a porous paper–based silver electrode was cut and placed with one end immersed in the LM trace at the designated connection site. A passive encapsulation layer of PDMS (15:1) was then applied, infiltrating the porous connector to form a robust and sealed interface. The other end of the connector was perforated and attached to a magnetic pin header on the PCB using LM paste, followed by PDMS encapsulation for mechanical stability. The skin-contact side of the patch was similarly equipped with porous connectors and covered with medical-grade adhesive gel.

The custom PCB incorporated a microcontroller (STM32L423KCU6), an AD8233 ECG sensor, an MPU-9250 inertial measurement unit (gyroscope and accelerometer), a voltage divider circuit for resistance measurement, a TransFlash card for local data storage, and a lithium-ion battery (500 mA·hour). All signals were sampled at 200 Hz.

### Device characterization

Surface morphology was analyzed using a JEOL-6390 scanning electron microscope. Electrical resistance changes were recorded with a Keithley 2612B source measure unit. Tensile tests for detection limits were conducted using an AERS G2 rheometer at 50 mm min^−1^. Cyclic tensile testing (10% strain, 5.2 × 10^5^ cycles) was performed using a custom translation stage at a speed of 350 mm s^−1^ and an acceleration of 200 mm s^−2^. In the simulated ECG measurements, Lead-II ECG waveforms (1-mV amplitude, 60-bpm rate) were applied to the device using an electrophysiological simulator (SKX-2000G, Xuzhou Mingsheng Electronic Technology).

A custom lung simulator was constructed by connecting a rubber balloon to a control PCB equipped with a JXFS-001 gas flow sensor, an XGZP pressure sensor, and a miniature vacuum pump. The operational protocol involved an initial pressure check: If pressure was detected, the system released gas. Otherwise, it initiated an inflation sequence to 3000 ml. After initial filling, the system performed 20 cycles of inflation/deflation (50 to 400 ml per step) at 5-s intervals. During testing, the patch was attached to the center of the balloon, and the resistance channel was connected to the source measure unit.

The 180-day stability test was performed to simulate realistic intermittent use. The device was periodically worn under two complementary conditions: (i) overnight for 8 to 12 hours, twice per week, during sleep and (ii) for 2 to 3 hours per day during routine activities (sitting, walking, etc.). Between wearing sessions, it was stored under ambient laboratory conditions (22 ± 2°C, 40 to 60% relative humidity). Performance measurements were taken at scheduled intervals after representative wearing sessions to ensure that the reported stability reflects real-world usage patterns.

### On-body testing

All studies involving human subjects were conducted in accordance with protocols approved by the Ethics Committee of the Hong Kong University of Science and Technology (approval no. HREP-2024-0274). Written informed consent was obtained from all participants. Before attachment, the skin was cleaned with alcohol wipes. The device was activated after adhesion of the medical-grade gel interface. The snoring measurement data were acquired from a voice recording software. The chest patch was positioned vertically along the midsternal line, with its upper edge ~10 cm below the clavicle and its U-shaped channel aligned parallel to the primary axis of chest expansion. The abdominal patch was placed parallel to the chest patch over the diaphragm region.

To ensure consistency and comparability while limiting intersubject and intra-activity variability, all cardiorespiratory measurements during daily activities were conducted under standardized conditions. Signals for sitting, working, talking, walking, and stair climbing were recorded directly during the performance of each activity to capture immediate physiological responses. The postsport measurement was acquired within 1 min after activity cessation while the subject remained seated at rest. Responses to mask wearing were recorded after a continuous wear period of 30 to 60 min, and the effect of caffeine intake was measured 1 to 2 hours after coffee consumption. These controlled time frames were implemented to ensure that the reported signals reliably reflect the intended physiological state for each condition.

### Signal processing

All training models were developed using Python (version 3.8) on the basis of a comprehensive dataset collected from eight volunteers. This dataset included various physiological signals, specifically ECG, accelerometer, gyroscope, and respiratory sensor signals and peripheral capillary SpO_2_. The sampling frequency for the ECG, accelerometer, gyroscope, and respiratory sensor signals was set at 200 Hz, ensuring a high temporal resolution for accurate physiological monitoring. In contrast, the SpO_2_ signal was recorded at a frequency of 12.5 Hz. The raw signals underwent a preprocessing phase, during which they were transformed into clinically relevant metrics, including the heart rate, heart rate variability, respiratory rate, tidal volume, and blood oxygen concentration. In addition, sleep posture was evaluated using a pretrained classification model, which enabled the accurate identification of different sleep positions based on the accelerometer data. The resulting processed signals were uniformly sampled at 1 Hz to facilitate further analysis. The preprocessed data were segmented using a sliding window approach. Each segment consisted of a 30-s window with a 20-s overlap between successive segments. This method allowed for the extraction of temporal features while maintaining enough segments for robust analysis.

The input layer receives a 6 by 30 two-dimensional data matrix, where each row represents a feature containing 30 processed feature values. These features include the heart rate, heart rate variability, respiratory rate, tidal volume, SpO_2_, and posture signals, comprehensively reflecting an individual’s physiological state. To capture the characteristic physiology of apnea-hypopnea events, which evolve over tens of seconds to minutes and exhibit delayed cross-modal interactions (e.g., respiratory decline followed by gradual oxygen desaturation and compensatory heart-rate changes), we adopted a three-layer bidirectional long short-term memory (BiLSTM) network. This architecture processes each window in both forward and backward directions, enabling precise event boundary detection by leveraging past and future context—a capability that standard long short-term memory lacks. Each BiLSTM layer contains 32 units and uses dropout (rate = 0.2) to improve generalization. The stacked layers first extract temporal patterns within individual modalities and then learn higher-order cross-modal relationships. The network concludes with a fully connected layer that outputs an event probability. The model was trained for 1000 iterations using the Adam optimizer (initial learning rate = 1 × 10^−4^) with binary cross-entropy loss, supervised by PSG-derived event annotations. A temporally aware weighted assessment mechanism was subsequently applied to finalize event detection and localization. This integrated pipeline, from robust feature engineering to a physiology-informed BiLSTM architecture, provides a computationally efficient framework for automated, multimodal apnea-hypopnea screening.

## Supplementary Materials

### The PDF file includes:

Tables S1 and S2 Figs. S1 to S28 Supplementary Notes S1 to S3 Legend for movie S1 References
