# Supplementary material for: Intermetallic-anchored epidermal EGaIn patch with analog constriction gates for cardiorespiratory monitoring
Source: Sci Adv. 2026 Jun 26;12(26):eaee5907. doi: 10.1126/sciadv.aee5907 (PMC13308624; doi:10.1126/sciadv.aee5907)
Supplement: Supplementary file 1 — Tables S1 and S2 Figs. S1 to S28 Supplementary Notes S1 to S3 Legend for movie S1 References [file sciadv.aee5907_sm.pdf]

Science Advances
AAAS

Supplementary Materials for

Intermetallic-anchored epidermal EGaIn patch with analog constriction gates for cardiorespiratory monitoring

Yue Li *et al.*

Corresponding author: Hnin Yin Yin Nyein, hnyein@ust.hk

Sci. Adv. 12, eaee5907 (2026)
DOI: 10.1126/sciadv.aee5907

The PDF file includes:

Tables S1 and S2
Figs. S1 to S28
Supplementary Notes S1 to S3
Legend for movie S1
References

Other Supplementary Material for this manuscript includes the following:

Movie S1

Table S1. The comparison of performance between our work and other advanced works.

	Detection Limit (%)	Linearity (R^2)	Hysteresis (%)	Cycling Stability	Range (%)	Ref.
Liquid metal-based sensor	0.4	0.976	1.902	500	150	(*49*)
	0.3	Nonlinear	0.11	3500	140	(*32*)
	0.04	Nonlinear	1	100000	140	(*35*)
	0.05	Nonlinear	7	8000	105	(*50*)
	0.1	Nonlinear	N/R	300	400	(*51*)
	1	0.99	N/R	200	20	(*52*)
	10	0.99	<0.1	10000	80	(*33*)
	1	0.98	0.448	3000	300	(*53*)
	N/A	Nonlinear	N/A	7500	110	(*54*)
	0.3	Nonlinear	N/A	300	0.5	(*55*)
	0.0033	Nonlinear	N/A	350	660	(*36*)
	0.01	0.998	0.4	>500000	700	This work
Carbon-based sensor	N/A	0.95	9.08	100	100	(*56*)
	N/A	Nonlinear	0.359	10100	500	(*57*)
	0.5	0.996	1.2	20100	70	(*58*)
	0.1	0.997	5	10000	50	(*59*)
	N/A	0.99	10	1000	60	(*60*)
	0.1	Nonlinear	2.3	10000	690	(*61*)
	N/A	0.996	12	100	100	(*62*)
Metal and polymer-based sensor	5	0.996	1	500	750	(*63*)
	N/A	Nonlinear	11.5	100	200	(*64*)
	N/A	0.98	1.5	2000	300	(*65*)
	1	0	2	500	2000	(*66*)
	0.75	0.998	8.61	10000	800	(*67*)
	2.9	Nonlinear	0.00179	1200	60	(*34*)

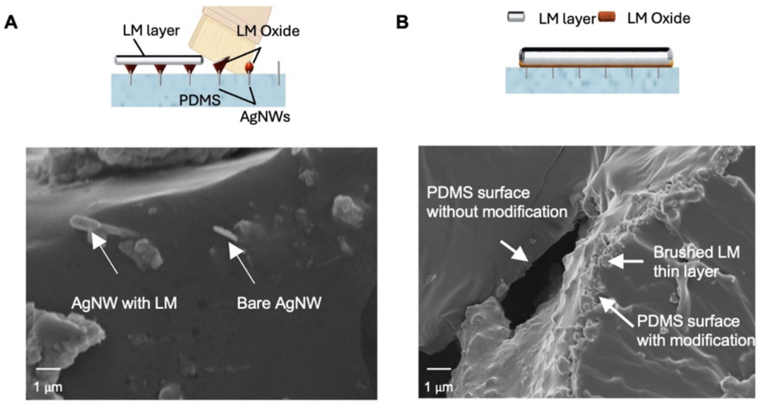

Fig. S1. Interfacial adhesion of LM with/without AgNW modification. SEM image showing LM preferentially adhering to (**A**) the protruding AgNWs, and (**B**) SEM demonstrating LM firmly adhering to the AgNW-modified PDMS interface (right) but readily detaching from the unmodified region (left) when sandwiched between two PDMS layers.

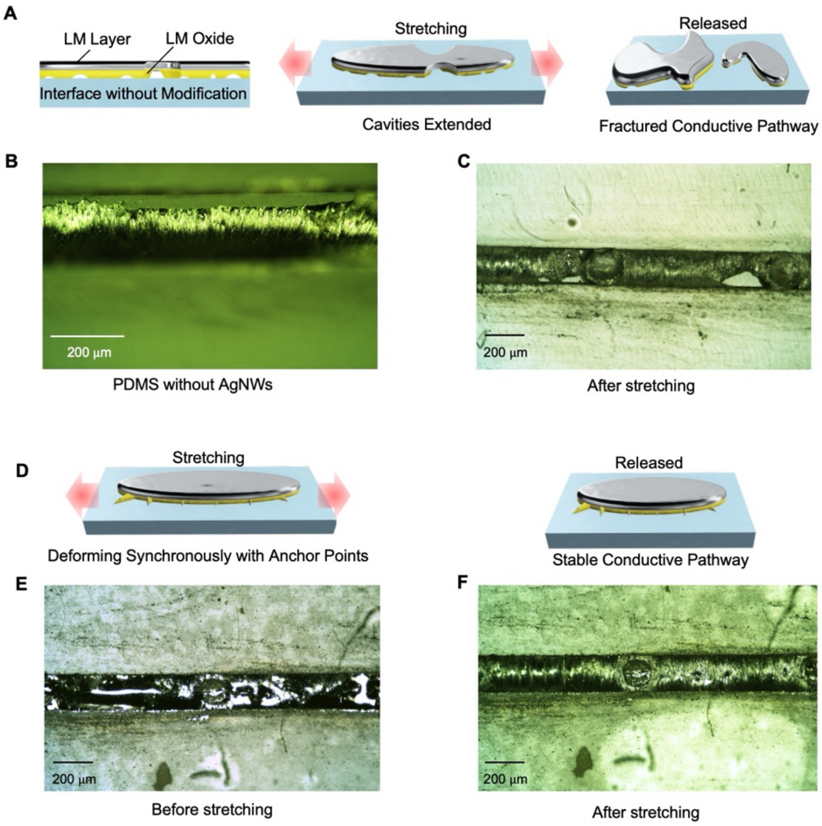

Fig. S2. Interfacial stability of the liquid-metal conductive pathway with and without AgNW anchoring. (**A**) Schematic illustration of the failure mechanism on an unmodified PDMS surface under mechanical deformation. Experimental observation of interfacial instability and cavity formation (**B**) before stretching and (**C**) after stretching. (**D**) Schematic illustration of the enhanced stabilization by the AgNW-modified system. (**E**) Enhanced liquid-metal adhesion achieved through AgNW anchoring. (**F**) Maintained conductive pathway integrity after repeated mechanical strain in the AgNW-modified system.

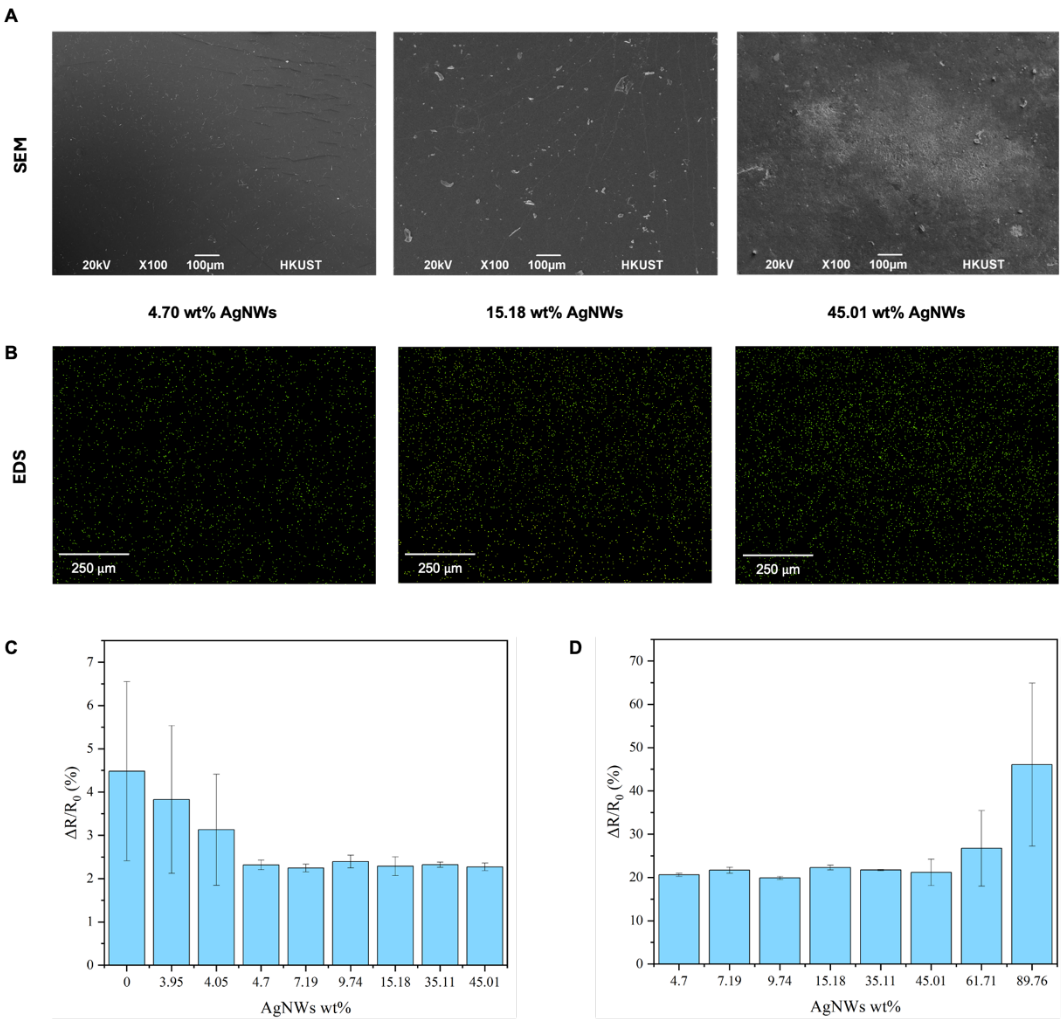

Fig. S3. Optimization of AgNW density for LM-PDMS interfacial anchoring. (**A**) SEM and (**B**) EDS images showing the density and distribution of AgNWs embedded within PDMS. (**C**) Resistance variation over 40 cycles at 2% strain across AgNW surface densities ranging from 0 to 45.01 wt%. (**D**) Resistance variation over 1,000 cycles at 20% strain across AgNW surface densities ranging from 4.70 to 89.76 wt%. Error bars represent the standard deviation of resistance values measured every 100 cycles from a single device over the full cycle range (n = 10).

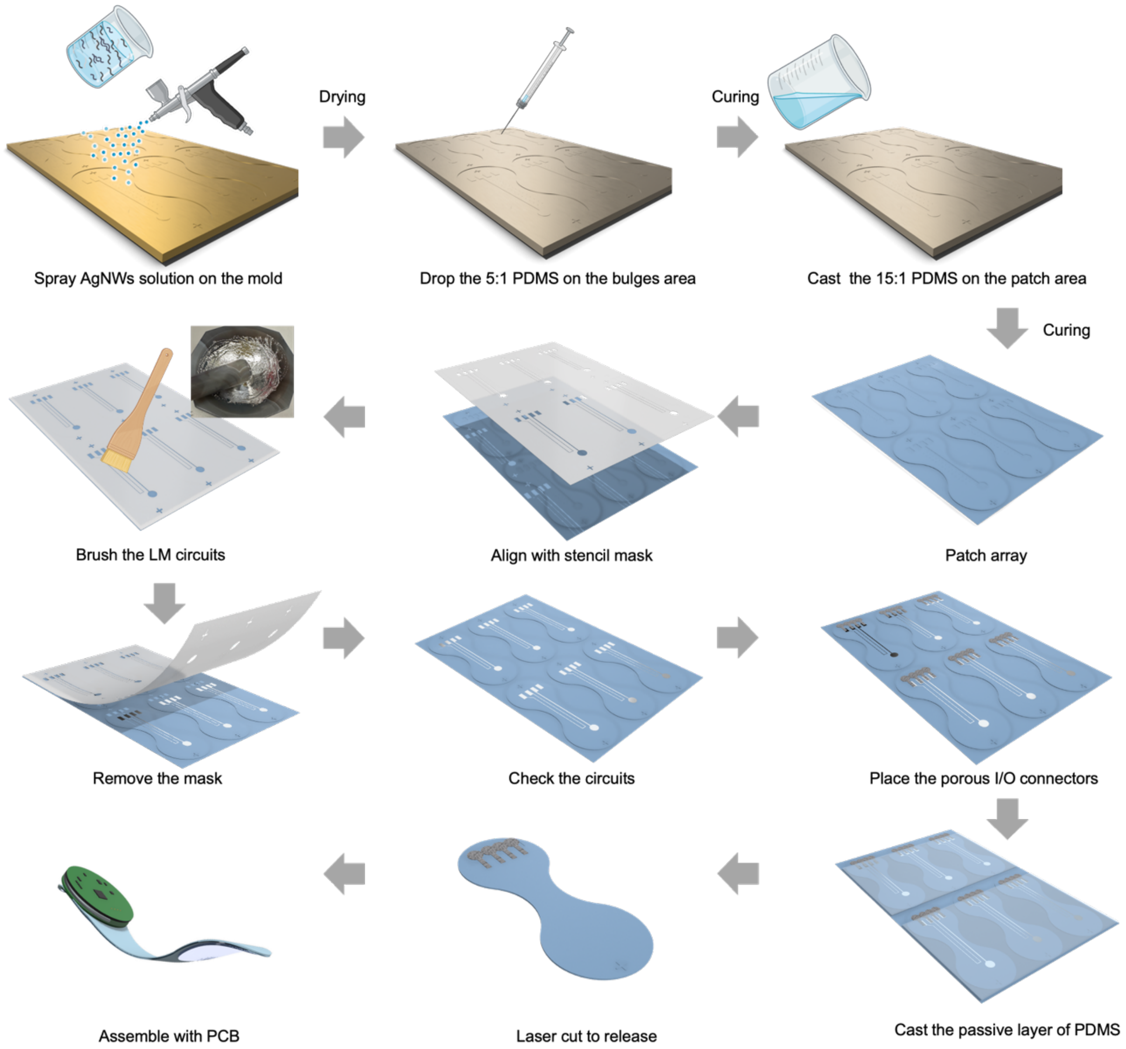

Fig. S4. Fabrication process flow of HELP.

Note S1. Design, Theoretical Analysis, and Numerical Simulation of the Graded Micro-Bulge Liquid-Metal Strain Sensor

To enhance the sensitivity of the strain sensor while maintaining high linearity, we engineered the patch with micro-bulge gates whose geometry and spatial distribution were systematically optimized. The bulges were made of a high–Young's modulus PDMS (base: curing agent = 5:1), whereas the surrounding patch substrate consisted of softer PDMS (base: curing agent =15:1). This modulus mismatch allowed the bulge structures to retain their shape under strain, locally amplifying deformation in the overlying liquid metal channel (*35*).

Experimental Design and Optimization of Micro-Bulge Gates

We designed micro-bulge gates with varying heights and densities to achieve gradual channel closure under stretching, thereby improving sensitivity and linearity. As illustrated in fig. S5, the bulge height decreased symmetrically from the center (experiencing the highest strain) toward the ends of the sensor. We compared several designs: Patch 0 (no bulges, control), Patch 1 (14 bulges of uniform height: 100 μm), Patch 2 (6 central bulges at 100 μm, peripheral ones at 99.5 μm), and Patch 3 (graded heights with three tiers, parameters in fig. S5). Among these, Patch 3 exhibited the best performance in both sensitivity and linearity (fig. S6 A-C).We further evaluated designs with reduced bulge density (7 bulges, Patches 4–6 corresponding to Patterns 1–3). Lower density not only diminished sensitivity enhancement but also impaired linearity. Comparative analysis in fig. S6 D confirmed that high-density patterning (e.g., Patch 3) outperforms low-density designs. The resistance responses of all designs with bulges (Patches 1–6) at 30% strain, compared to the control (Patch 0), are shown in Fig. S7.

To establish a theoretical foundation for the graded hemispherical gate design and to demonstrate its design tunability and generalizability, we developed a complementary analytical and numerical framework. The analytical model provides mechanistic insight into how gate radius dispersion and density govern sensor performance, while the numerical simulation based on Hertz contact theory validates the model and enables quantitative exploration of the design space.

S1.1. Analytical Model of Gate Activation and Resistance Response

To understand why the graded design outperforms uniform arrays, we developed a semi-analytical model grounded in elastic geometry and contact mechanics.

S1.1.1. Baseline Resistance-Strain Relationship

For a LM channel of length L, and cross-section A, resistance is given by $R = \rho\frac{L}{A}$ where ρ is resistivity. Under uniaxial tensile strain ε, the channel length becomes $L(\varepsilon) = L_0(1 + \varepsilon)$ where L_0 is initial length. Assuming near-incompressibility of PDMS (Poisson ratio close to 0.5), volume conservation gives $L(\varepsilon)\,A(\varepsilon) \approx L_0 A_0$ where A_0 is the initial cross-sectional area *(10)*. Therefore, the baseline resistance without gate constriction is:

$$R_{base}(\varepsilon) = \rho \frac{L_0(1+\varepsilon)}{A_0/(1+\varepsilon)} = R_0(1+\varepsilon)^2 \quad \text{(S1)}$$

where R_0 is the initial resistance. A correction factor k_{base} is introduced to account for non-ideal effects. Eqn. S1 normalizes the baseline resistance to its initial value, with $k_{\text{base}} = 1$ corresponding to the ideal geometric scaling from Eq. S5.

$$\frac{R_{\text{base}}(\varepsilon)}{R_{\text{base}}(0)} = 1 + k_{\text{base}}[(1+\varepsilon)^2 - 1] \quad \text{(S2)}$$

This equation captures the natural resistance increase due to geometric stretching of the channel.

S1.1.2. Gate Activation Mechanics

Let h be the effective channel height above each gate (the initial gap between the gate top and the channel ceiling), and global axial strain ε induces vertical displacement δ (ε) (compression) of the channel:

$$\delta(\varepsilon) = \beta h\, \varepsilon \quad \text{(S3)}$$

where β is the effective structural coupling coefficient that describes how efficiently horizontal stretch converts to vertical compression; it depends on the PDMS modulus and channel geometry.

Let r_i denote the radius of the ith gate. We define ε_i as the global activation strain – the applied strain at which this gate first contacts the channel ceiling. Contact occurs when the vertical displacement equals the initial gap between the gate top and the channel ceiling:

$$\beta h \varepsilon_i = h - r_i \quad \text{(S4)}$$

Solving gives:

$$\varepsilon_i = \frac{h - r_i}{\beta h} \quad \text{(S5)}$$

Therefore, larger radii correspond to smaller activation strain, producing earlier contact. As the strain distribution along the channel is non-uniform, with higher strain near the center, the local strain at the position of gate i^{th} is:

$$\varepsilon_{\text{local},i} = g_i \varepsilon \quad \text{(S6)}$$

where g_i is a position-dependent factor (ranging from $g_{center} > 1$ to $g_{edge} < 1$) that accounts for strain concentration toward the center. The activation level of each gate follows a logistic function, capturing the smooth transition from no contact to full constriction:

$$s_i(\varepsilon) = \frac{1}{1 + \exp\left(-\frac{\varepsilon_{\text{local},i} - \varepsilon_i}{\Delta}\right)} \quad \text{(S7)}$$

where s_i ranges from 0 to 1 and Δ controls transition sharpness. A smaller Δ means the gate activates more abruptly. The total constriction effect is the weighted sum of individual gate activations. Larger gates contribute more to resistance change because they constrict a wider portion of the channel. We, therefore, weight each gate by its radius:

$$Q(\varepsilon) = \sum_{i=1}^{N} \frac{r_i}{\bar{r}}\, s_i(\varepsilon) \tag{S8}$$

where $\bar{r}$ indicates the mean radius, and N is the number of gates.

S1.1.3. Role of Radius Dispersion and Gate Density

From Eq. S5, the activation strain decreases linearly with radius. Therefore, the spread of activation strains is proportional to how much the gates' radii vary:

$$\sigma_\varepsilon \propto \frac{\sigma_r}{\bar{r}} \tag{S9}$$

where σ_r is the standard deviation of gate radii. This indicates that a larger radius dispersion spreads activation events over a wider strain range – some gates activate very early (large radii), others very late (small radii). This extends the responsive range and improves linearity.

To quantify how radius dispersion affects sensitivity, we introduce a gradient scaling factor:

$$S_{gradient} = 1 + \eta \frac{\sigma_r}{\bar{r}} \tag{S10}$$

where η represents an amplification factor determined by scaling to experimental data. Eqn. S10 tells us that when $\sigma_r = 0$ (all gates identical), $S_{gradient} = 1$, meaning no enhancement from grading. As radius dispersion increases, $S_{gradient}$ increases, reflecting the benefit of spreading activation events.

The total constriction effect also scales with the number of gates. We introduce a density scaling factor:

$$S_{density} = \frac{N}{N_{full}} \tag{S11}$$

where N_{full} is the number of gates in the full-density configuration ($N_{full} = 14$ in our experimental design.) This factor represents the fraction of gates relative to the full design. For example, reduced-density patches of $N = 6$ results in $S_{density} = 0.43$, indicating approximately 43% of the constriction effect of the full-density design. Hence, more gates provide more constriction events, increasing overall sensitivity. Fewer gates reduce sensitivity proportionally.

The overall constriction effect combines both factors:

$$S_{total} = S_{gradient} \cdot S_{density} = \frac{N}{N_{full}} \left(1 + \eta \frac{\sigma_r}{\bar{r}}\right) \tag{S12}$$

This total scaling factor multiplies the constriction effect in Eqn S8 to give the overall resistance change. The normalized resistance can then be expressed as:

$$\frac{R(\varepsilon)}{R(0)} = \frac{R_{\text{base}}(\varepsilon)}{R_{\text{base}}(0)} \cdot \exp(S_{total} \cdot \mathrm{Q}(\varepsilon)) \quad \text{(S13)}$$

where the exponential form ensures smooth transitions and positivity.

S1.1.4. Model Limitations

The logistic function was chosen because contact formation in soft elastomeric structures is inherently continuous: contact initiates locally, expands nonlinearly with increasing strain, and asymptotically approaches saturation. The present model provides a reduced-order description of the sensor's electromechanical response. It captures the dominant strain–resistance trends through geometrically informed deformation and strain-activated contact growth but does not explicitly resolve full spatial stress distributions or time-dependent effects such as viscoelasticity and cyclic fatigue. These simplifications reflect the goal of establishing a physically interpretable and computationally tractable framework, while acknowledging that more detailed analyses would be required for higher-order dynamic effects.

S1.1.5. Summary and Design Implications

The analytical model reveals that sensor performance is governed by two key parameters derived directly from gate geometry. The first is radius dispersion, $\frac{\sigma_r}{\bar{r}}$, which controls the spread of activation strains across the channel. A larger dispersion radius improves linearity and extends the overall sensing range. The second is gate density, $\frac{N}{N_{full}}$, a parameter scales the total constriction effect, with a higher density yielding greater sensitivity.

These parameters can be independently tuned for different applications. Increasing radius dispersion $\frac{\sigma_r}{\bar{r}}$ spreads activation events across the strain range, enhancing linearity – ideal for applications requiring accurate waveform reconstruction. Increasing gate number N adds more constriction events, boosting sensitivity – ideal for detecting subtle respiratory efforts. The optimal design balances both by using moderate radius dispersion with full gate density. This tunability demonstrates that the graded gate concept is generalizable: by adjusting $\frac{\sigma_r}{\bar{r}}$ and *N*, the sensor can be tailored for different applications while the underlying physics remains unchanged.

S1.2.1. Numerical Simulation and Design Optimization Based on Hertz Contact Theory

Based on the analytical model, we established that higher gate density and larger radius dispersion lead to improved sensitivity and broader responsive range. To identify the optimal design boundaries and validate the saturation limit of the gradient effect, we employed a numerical simulation based on Hertz contact theory for quantitative confirmation.

We assume the 5:1 PDMS gates are rigid. During the stretching process, the 15:1 PDMS substrate comes into contact with the PDMS gates, compressing the liquid metal (LM) channel and changing its resistance (Fig. S8). Since the gates are distributed in two symmetrical columns, we simulated the response of half a column.

For the reduced-density configurations (Patches 4–6, corresponding to Modes 4–6 below), the design nominally contains 7 gates total (3.5 per column). Because the gates are discrete, we rounded to 3 gates per column (6 total) for simulation. Given the small difference in gate count and the dominant role of the radius gradient, this simplification does not alter the qualitative conclusions regarding reduced-density performance.

We defined ten configurations (Modes 0-9) mirroring the experimental patches:

Mode 0: No gates (control)

Mode 1: Uniform radii [100, 100, 100, 100, 100, 100, 100] μm (7 gates per column, 14 gates total)

Mode 2: Slight gradient [100, 100, 100, 99.5, 99.5, 99.5, 99.5] μm (7 gates per column, 14 gates total)

Mode 3: Moderate gradient [100, 99.5, 99.5, 99.5, 99, 99, 99] μm (7 gates per column, 14 gates total)

Mode 4: Uniform radii, 7 gates total [100, 100, 100] μm (3.5 gates per column, simulated as 3 gates with scaling to account for the half gate)

Mode 5: Slight gradient, 7 gates total [100, 99.5, 99.5] μm (3.5 gates per column, simulated as 3 gates with scaling to account for the half gate)

Mode 6: Steep gradient, 7 gates total [100, 99.5, 99] μm (3.5 gates per column, simulated as 3 gates with scaling to account for the half gate)

Mode 7: Enhanced gradient, 7 gates [100, 99, 99, 99, 98, 98, 98] μm (predictive, 7 gates per column, 14 gates total)

Mode 8: Increased density, 10 gates [100, 100, 99, 99, 99, 99, 98, 98, 98, 98] μm (predictive, 10 gates per column, 20 gates total)

Mode 9: Increased density, 10 gates [100, 100, 99.5, 99.5, 99.5, 99.5, 99, 99, 99, 99] μm (predictive, 10 gates per column, 20 gates total)

Modes 1–3 correspond to Patches 1–3 (full density, 14 gates total, 7 per column). Modes 4–6 correspond to Patches 4–6 (reduced density, 7 gates total, 3.5 per column). Because the gates are discrete, we simulated 3 gates per column and applied appropriate scaling to account for the half gate; given the small difference in gate count and the dominant role of the radius

gradient, this simplification does not alter the qualitative conclusions. Modes 7–9 are predictive configurations explored to further probe the design.

S1.2.2. Hertz Contact and Resistance Calculation

For each gate, the pressure distribution is calculated based on Hertz contact theory *(68)*. The Hertz contact radius and pressure distribution are given by:

$$a_{hertz} = (\frac{3F^2}{4E^2R})^{1/3} \quad (68) \tag{S14}$$

where a_{hertz} is the contact radius (m), F is the contact force (N), R is the gate radius (m), and E is the elastic modulus (Pa) of 15:1 PDMS.

The pressure distribution within contact region is:

$$p(r) = \frac{3F}{2\pi a^2}\sqrt{1-(\frac{r}{a})^2} \quad (68) \tag{S15}$$

where $p(r)$ is the contact pressure at radial distance from the contact center r (Pa). The liquid metal thickness under compression is approximated using a simplified linear elastic analogy:

$$h_{LM}(r) = h_0 - \frac{p(r)}{E_{LM}} \quad (69) \tag{S16}$$

Where $h_{LM}(r)$ is the liquid metal thickness at radial distance r, h_0 = 3 μm is the initial thickness, and E_{LM} is the effective modulus of liquid metal. While liquid metal is a fluid rather than an elastic solid, this approximation provides a computationally tractable way to capture the trend of thickness reduction under contact pressure without requiring full fluid dynamics modeling. For points where $h_{LM}(r) < 1$ μm, the LM is considered fully displaced and non-conductive at that location.

The resistance calculation employs a two-dimensional discretization method in which the contact region under each gate is divided into an 800×800 grid where j indexes the columns (along the channel width) and k indexes the rows (along the channel length). For each column j, the column resistance is calculated as:

$$R_{\mathrm{col},j} = \frac{\rho \cdot \Delta x}{\sum_k h_{\mathrm{LM},jk} \cdot \Delta y} \tag{S17}$$

where ρ is the resistivity of liquid metal, Δx and Δy are the grid spacings, $h_{\mathrm{LM},jk}$ is the liquid metal thickness at grid point (*j*, *k*). Finally, the total resistance is obtained by summing the resistances of total column N in series:

$$R_{\mathrm{total}} = \sum_{j=1}^{N_{\mathrm{col}}} R_{\mathrm{col},j} \tag{S18}$$

S.1.2.3. Stress Distribution Along the Channel

Based on the patch geometry, the tensile stress is not uniformly distributed along the channel. The center of the patch experiences greater strain than the edges during stretching, meaning that gates near the center are subjected to higher compressive forces. To account for this in the simulation, we applied transfer exponential coefficients calibrated to match the simulated overall resistance to experimental curves: 1.03 per successive gate for the full-density configuration (28 gates total, 14 per column), 1.07 for the reduced-density configuration (14 gates total, 7 per column), and 1.02 for the higher-density predictive configurations (40 gates total, 20 per column). These coefficients progressively increase the force applied to each gate moving from the edge toward the center, capturing the non-uniform strain distribution.

S1.2.4. Simulation Results

The simulation results, plotted in Fig. S8, show that graded radii configurations outperform uniform designs. Mode 3 exhibits the highest sensitivity, confirming that an optimal balance between radius dispersion and gate density is essential for maximizing performance.

Reduced-density designs generally exhibit lower sensitivity than their full-density counterparts, confirming that gate density is a critical factor. However, Mode 6 achieves sensitivity comparable to full-density graded designs, suggesting that a steeper gradient can partially compensate for lower gate density.

Further increasing the radius gradient while keeping density constant does not improve performance; Mode 7 exhibits lower sensitivity than Mode 3. This occurs because the gradient and gate density must be matched. With the same gate density as Mode 3, the steeper gradient in Mode 7 creates a mismatch: the smallest gates activate only at very high strains, leaving insufficient constriction capacity to enhance overall sensitivity.

To explore the trade-off between gate density and radius gradient, we extended the simulation to higher-density configurations. Mode 8 shows sensitivity close to Mode 3, but Mode 9 does not outperform Mode 3. This suggests that simply increasing density without optimizing the gradient does not guarantee improved performance. These results confirm that an optimal balance between gate density and radius dispersion is essential, and why Mode 3 was selected as the optimal configuration.

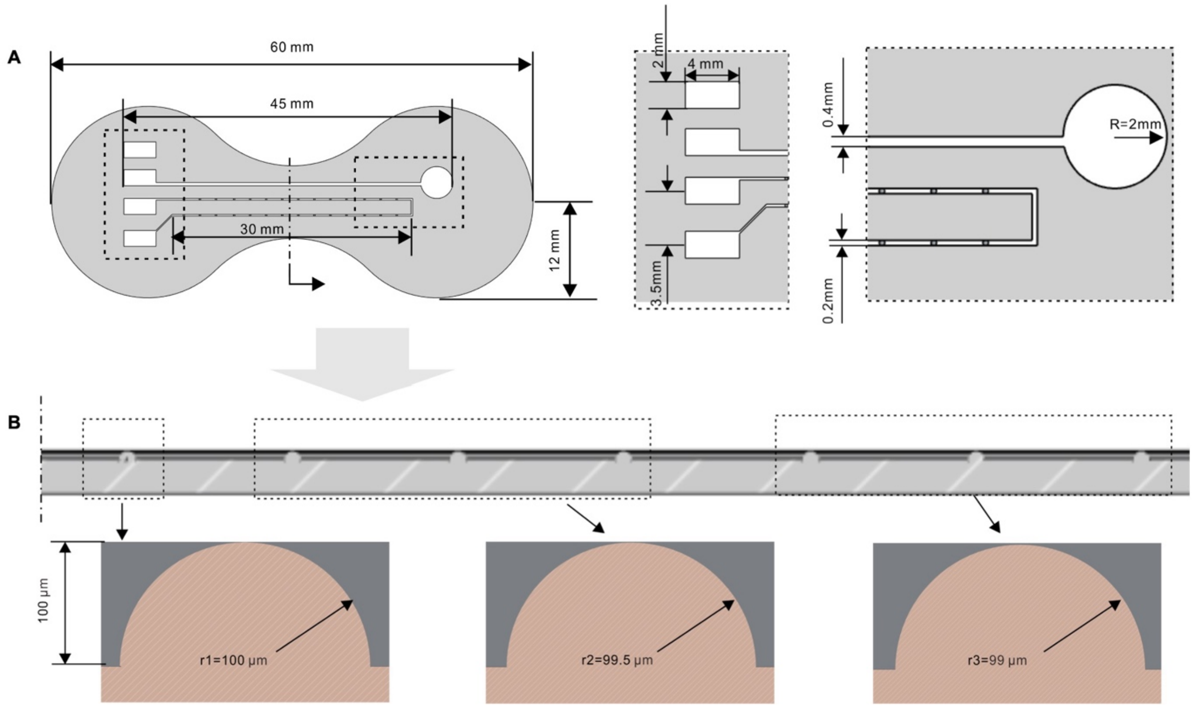

Fig. S5. Geometric design parameters of the patch architecture and micro-bulge gates. (**A**) Key dimensions and layout of the patch and microchannel design. (**B**) Cross-sectional schematic illustrating the distribution and diameter profile of the micro-bulges within the channel.

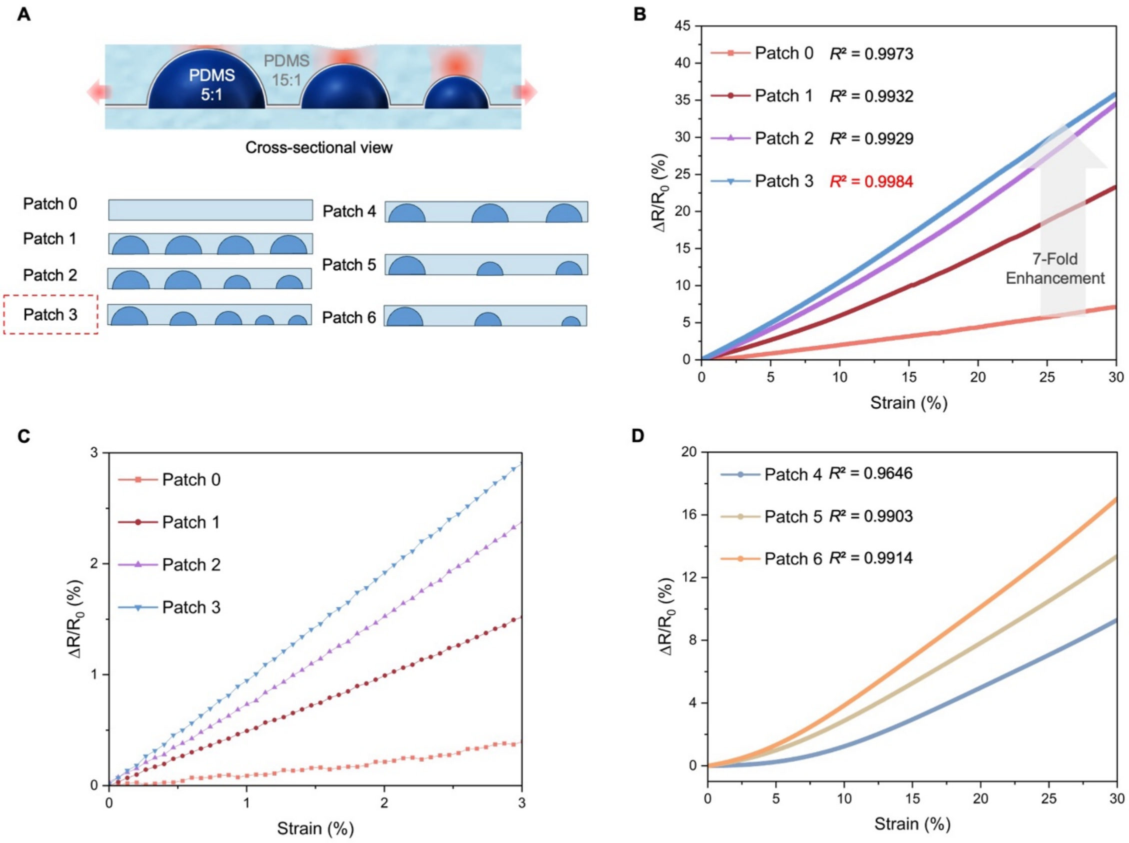

Fig. S6. Resistance response and linearity under strain for different micro-bulge gate designs. (**A**) Schematic cross-sections of the microchannel with micro-bulge gates for Patch designs 0–6. (**B**) Relative resistance change as a function of applied strain with linearity comparison across Patch 0–3. (**C**) Resistance variation under small strains (<3%), representing weak respiration, for the four designs. (**D**) Performance comparison of designs with reduced bulge density (Patch 4-6).

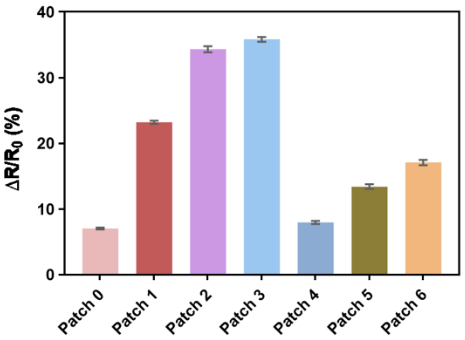

Fig. S7. Comparison of resistance responses at 30% strain between the control patch (Patch 0, without bulges) and all patch designs incorporating hemispherical bulges (Patches 1–6). Error bars indicate standard deviation of sensors (*n* =10).

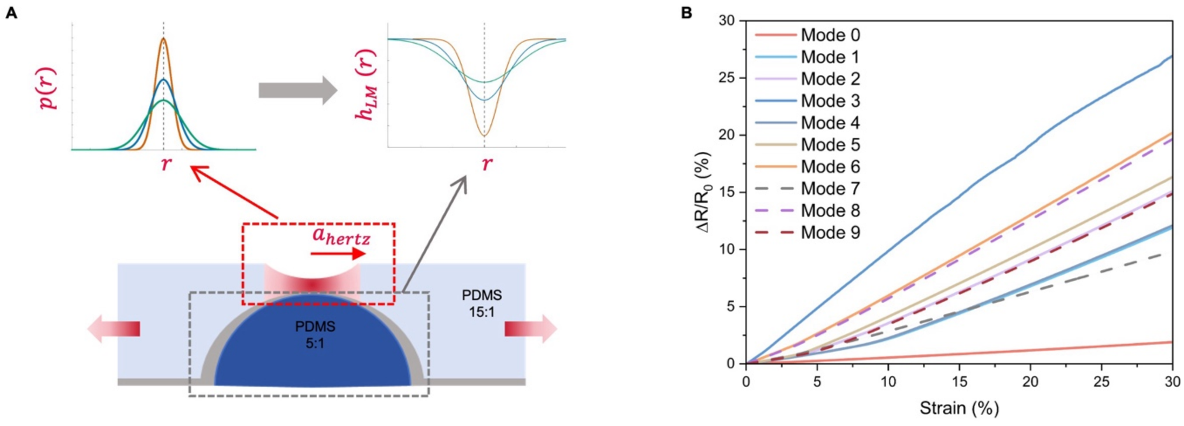

Fig. S8. Numerical simulation of resistance–strain response for different micro-bulge configurations. (**A**) A schematic diagram of stress distribution and LM thickness variation during the tensile process. a_{hertz} represents the contact radius, r is the radial distance of the LM layer from the PDMS gate plane center, $p(r)$ is the contact pressure at radial distance from the contact center *r*, and $h_{LM}(r)$ is the liquid metal thickness at radial distance *r*. (**B**) The normalized resistance change $(\Delta R/R_0)$ is plotted as a function of applied strain.

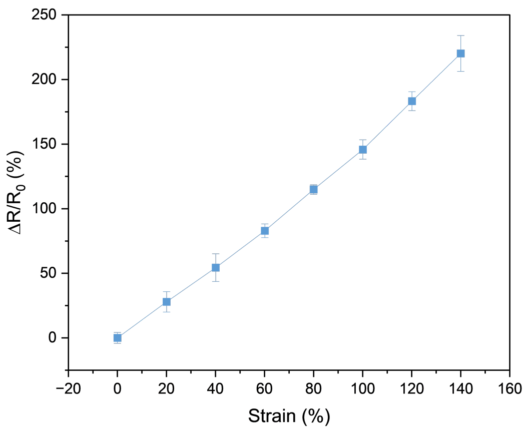

Fig. S9. Relative resistance change of Patch 3 under applied strains ranging from 0% to 140% (*n* =3).

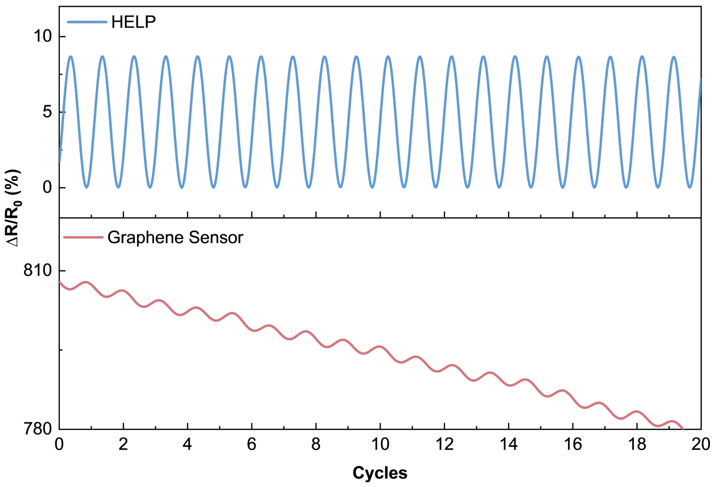

Fig. S10. Comparison of resistance recovery performance between HELP and a commercial graphene sensor. Due to energy loss from internal friction, the conductive filler of the graphene sensor separates upon loading but does not fully re-aggregate upon unloading, creating a hysteresis effect.

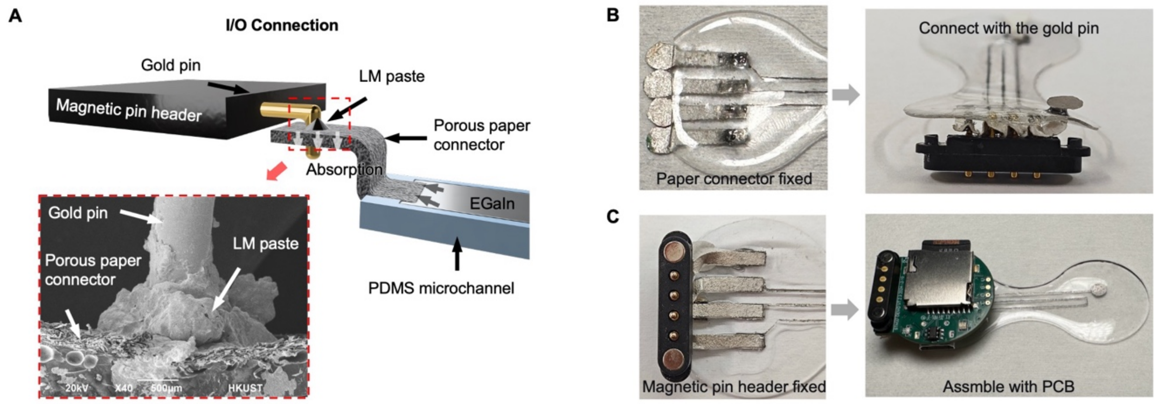

Fig. S11. Structural and functional details of the paper-based porous I/O connector. (**A**) Schematic of the connector providing a stable electrical and mechanical interface between the EGaIn trace and a gold pin, with the inset SEM image detailing the connection interface. (**B**) The encapsulated porous connector on the patch (left), followed by the attachment of the mating magnetic pin header (right). (**C**) The corresponding PCB module with a magnetic connector for easy assembly with the patch.

Note S2. Mechanism of overshoot reduction via the micro-bulge structure

Due to the incompressibility of LM, the deformation of the microchannel under strain induces a restoring pressure from the LM against the constricted PDMS walls, effectively widening the channel and resulting in a transient drop in electrical resistance (*27*). This phenomenon produces a significant overshoot in the resistance response during dynamic loading and, under cyclic conditions, leads to pronounced signal distortion and hysteresis (*34*). The introduction of the three-dimensional micro-bulge structures effectively counteracts this effect by redistributing the LM-induced pressure in the horizontal direction, thereby substantially mitigating the overshoot and significantly improving the hysteresis behavior in the electromechanical response of the sensor (fig. S12) (*26*).

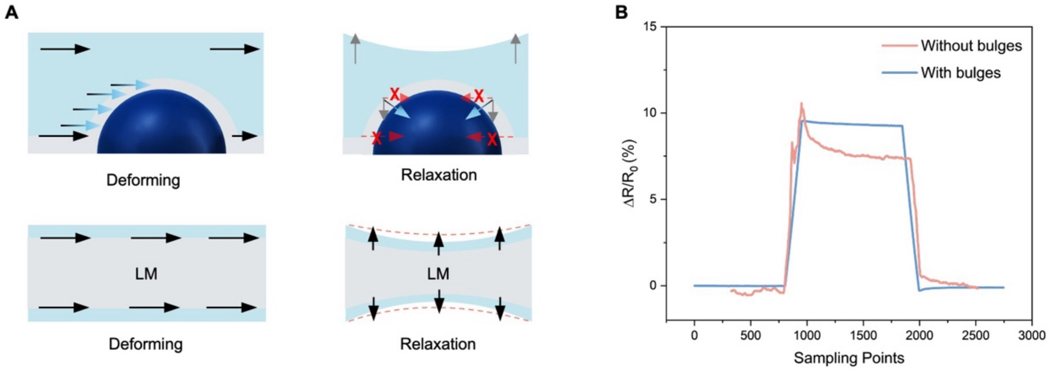

Fig. S12. Mechanism of overshoot reduction via the micro-bulge structure. (**A**) Schematic pressure distribution of the LM on the PDMS surface with and without micro-bulges during the stretching and relaxation process. (**B**) Significant overshoot of resistance observed in the design without micro-bulges compared to the bulge-enhanced design.

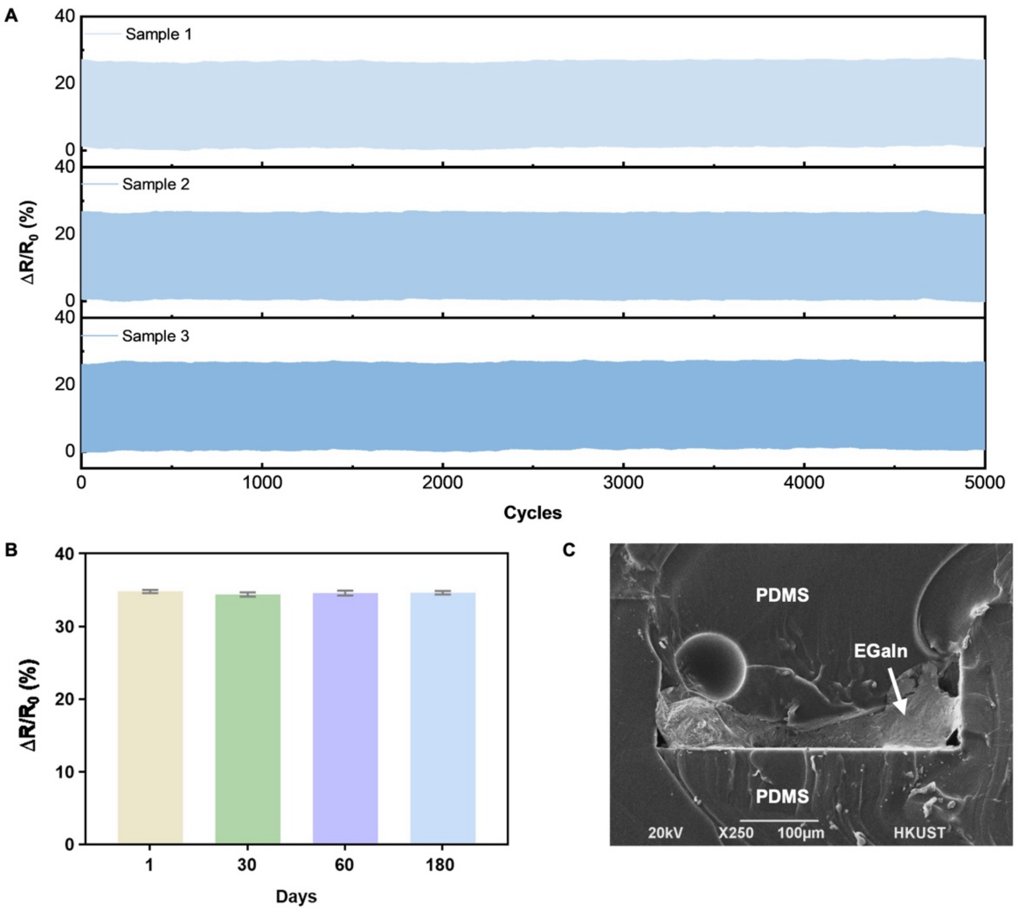

Fig. S13. Long-term stability of the strain-sensing performance of HELP. (**A**) Resistance response of three different HELP devices under 5000 cyclic stretching cycles (20% strain), demonstrating reproducible mechanical-electrical durability across replicates. (**B**) The resistance values measured repeatedly at 30, 60, and 180 days remained comparable to those on day 1 (n = 10). (**C**) Cross-sectional SEM image of a HELP patch after 180 days of storage and intermittent use, confirming the intact PDMS encapsulation with no leakage or migration of the encapsulated liquid metal or silver nanowires.

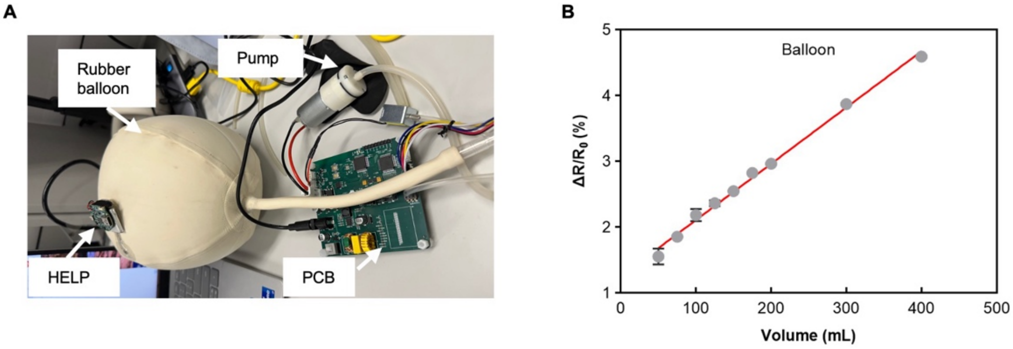

Fig. S14. Bench-top characterization of the sensor’s volumetric response using a balloon-based simulator. (**A**) Photograph of the respiratory simulation setup. (**B**) Linear correlation between the relative change in resistance of the HELP sensor and the preset volume change.

Note S3. Tidal Volume Calibration Protocol

The sensor was first characterized using a custom balloon-based lung simulator (Fig. S14A), which provides precise volumetric displacement (50–400 mL) under controlled conditions. This established the baseline relationship between applied strain and air volume, confirming a linear response with a resolution of 25 mL (Fig. S14B).

To validate the sensor under realistic conditions and assess inter-subject variability, ten healthy adults (5 male, 5 female) with diverse body morphologies were recruited. Participant characteristics are summarized in Table S2, with BMI ranging from 18.7 to 27 $kg \cdot m^{-2}$ and neck circumference from 29.5 to 41.5 cm.

Each participant wore HELP patches on the chest and abdomen. Respiratory strain signals were recorded simultaneously with airflow measured by a commercial spirometer (Model LK-T2016, Lingkang Electronic Technology Co., Ltd., China). Participants performed three repetitions each of normal, medium, and deep inhalations, targeting volumes of 500–5000 mL guided by visual feedback from the spirometer. A 10-second breath-hold preceded each maneuver to clearly demarcate individual breaths.

A strong linear correlation was observed across all individuals ($R^2 = 0.966$), as shown in Fig. S15. The relationship is described by:

$$V\,(\mathrm{mL}) = 29.66 \times \Delta R/R_0 + 78.36$$

This confirms the linear response of HELP across individuals and provides a straightforward method for personalized tidal-volume calibration.

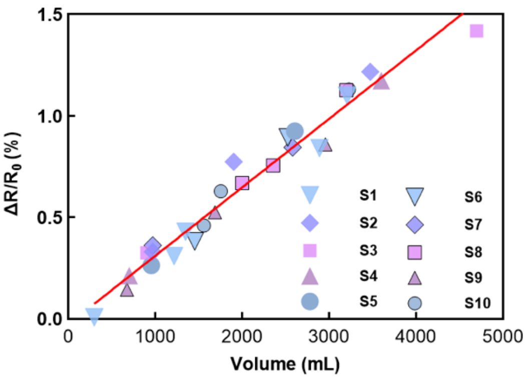

Fig. S15. Validation of the resistance-to-tidal-volume model during on-body testing. Linear relationship between the HELP signal and spirometer-measured volume in 10 human subjects (S1 – S10), confirming model performance and capturing inter-subject variability under realistic thoracic biomechanics.

Table S2 Anthropometric data for the inter-subject variability study.

Subject Number	Age	Sex	BMI ($kg \cdot m^{-2}$)	Neck circumference (cm)
S1	33	Female	26	36.5
S2	33	Male	22	41
S3	27	Male	19	36.5
S4	29	Male	22.4	38
S5	26	Male	27	40
S6	23	Male	26.8	41.5
S7	26	Female	26	33.5
S8	27	Female	19	31
S9	28	Female	18.7	29.5
S10	37	Female	19	30.5

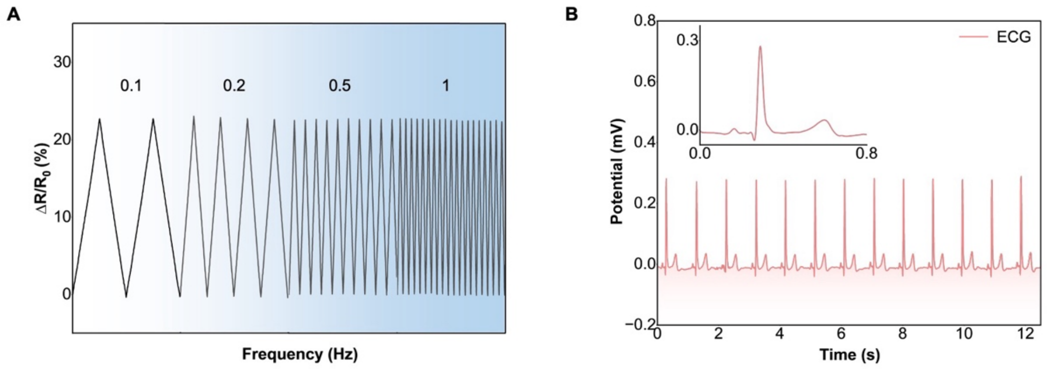

Fig. S16. Performance of the HELP system under stretching frequencies from 0.1 to 1 Hz at 20% tensile strain. (**A**) Stable cyclic resistance response. (**B**) Stable ECG signal.

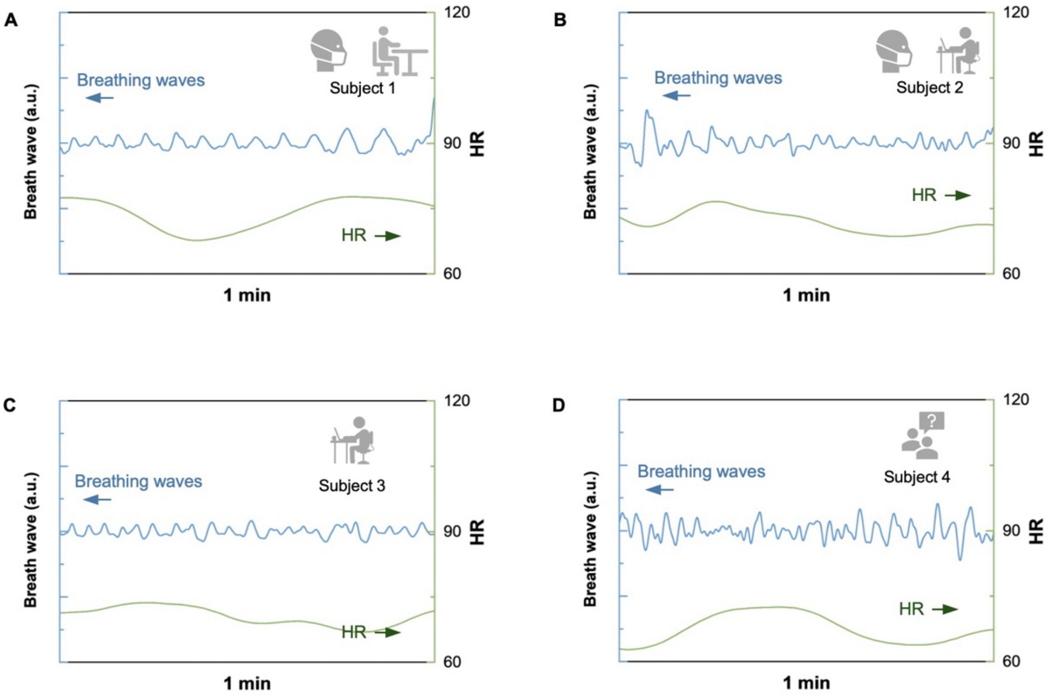

Fig. S17. One-minute respiratory (inferred airflow volume overlaid) and concurrent ECG from four subjects under different activities: (**A**) Subject 1 wearing a mask while sitting still; (**B**) Subject 2 wearing a mask while working; (**C**) Subject 3 working; (**D**) Subject 4 talking.

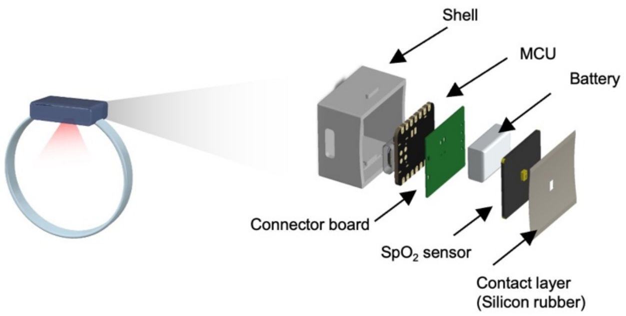

Fig. S18. Exploded view of the components in the reflective PPG-based SpO_2 ring.

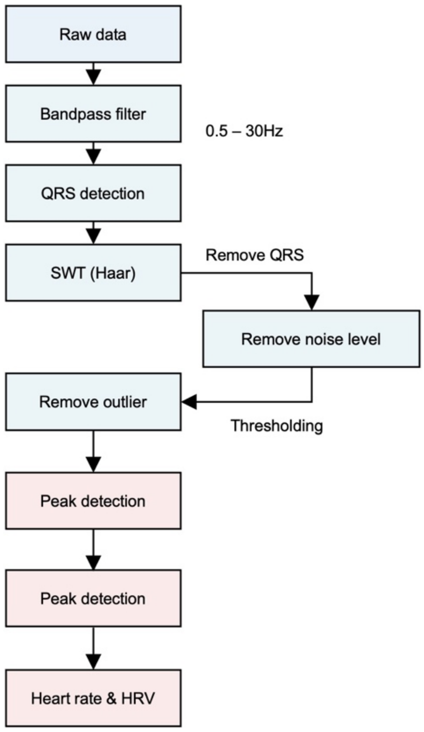

Fig. S19. Workflow for ECG data processing. The process includes: (1) bandpass filtering (0.5-30 Hz) to remove noise and baseline drift, (2) QRS complex detection and waveform characterization, (3) Stationary wavelet transform (SWT) for advanced noise suppression, (4) R-peak detection for heart rate calculation, and (5) heart rate variability (HRV) analysis through temporal analysis of successive R-R intervals.

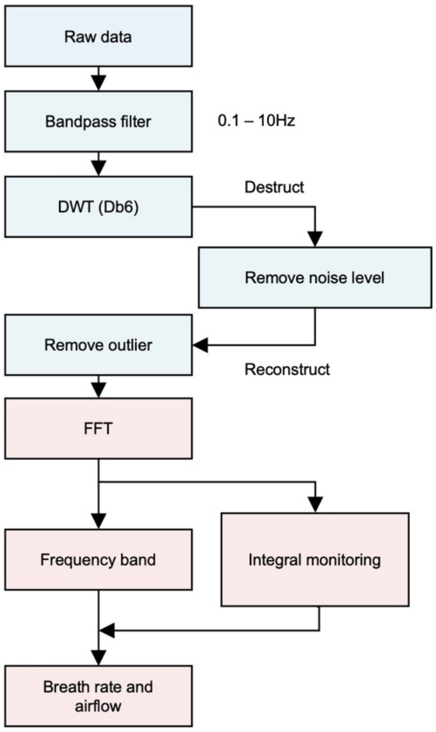

Fig. S20. Workflow for respiratory data processing. Schematic diagram illustrating the step-by-step computational pipeline for extracting respiratory parameters from raw signals. The process includes: (1) applying a 0.1-10 Hz bandpass filter to remove non-respiratory components; (2) performing discrete wavelet transform (DWT) for noise reduction; (3) using Fourier transform to identify fundamental respiratory frequency; (4) calculating respiratory volume through integration of the processed waveform; and (5) outputting continuous respiratory rate, tidal volume estimates, and waveform characteristics for further analysis.

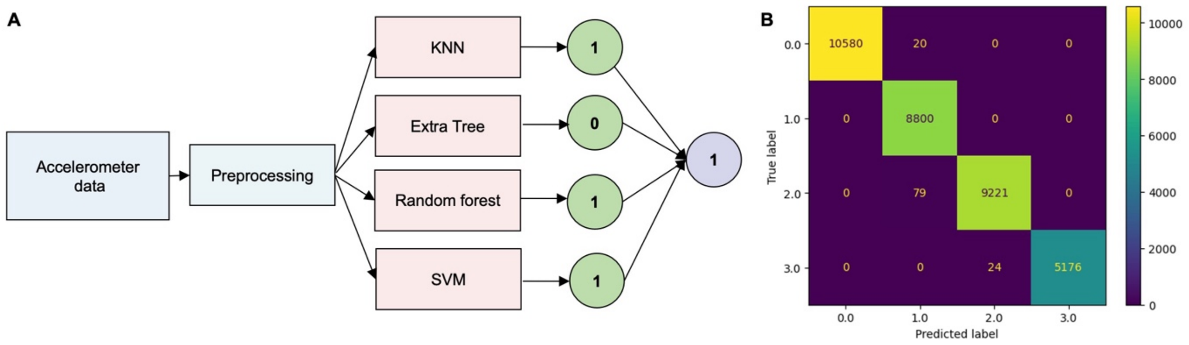

Fig. S21. Sleep position recognition model and performance evaluation. (**A**) Schematic of the sleep posture recognition method combining K-Nearest Neighbors (KNN), Extra Trees, Random Forest, and Support Vector Machine (SVM) algorithms through a weighted ensemble approach. (**B**) Confusion matrix showing the classification performance across four posture categories, with an overall accuracy of 99.54%.

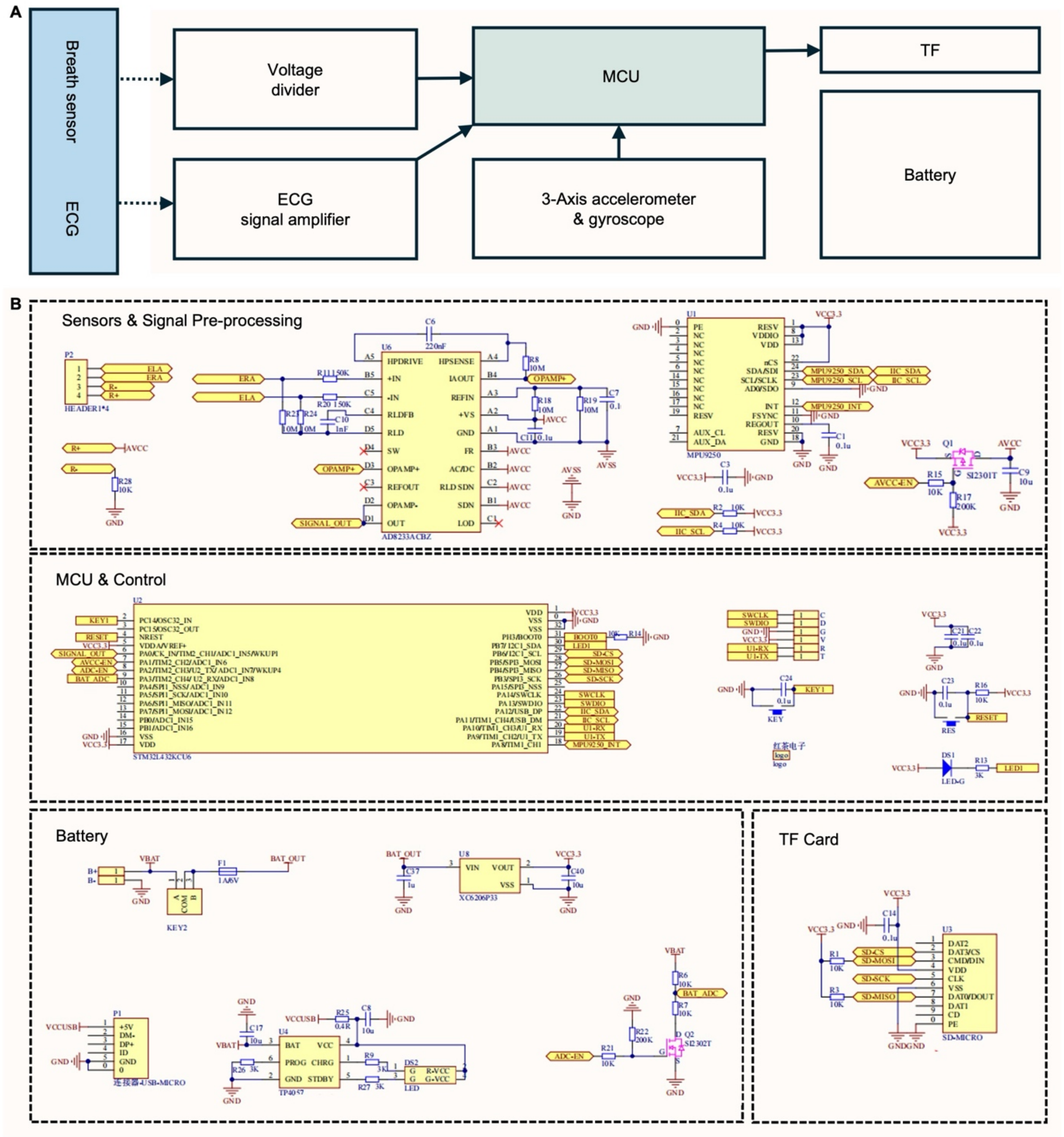

Fig. S22. PCB design of the HELP system. (**A**) Overall system design layout. (**B**) Schematic illustrating the integration of the sensor, signal pre-processing, MCU, control, power management module, and data storage interface (TF card).

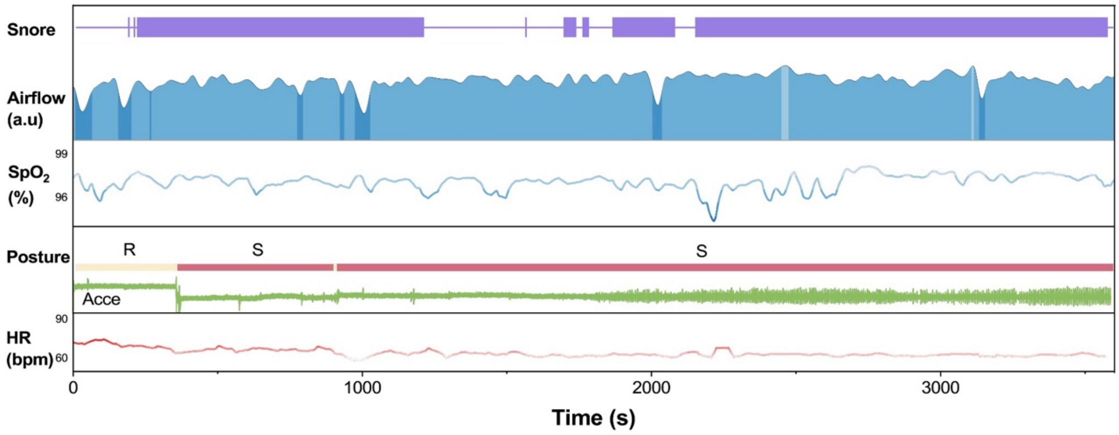

Fig. S23. An example of overnight sleep monitoring with HELP showing a hypopnea event. Multi-parameter physiological signals recorded during extended sleep monitoring, illustrating a hypopnea event occurring at approximately 2000 seconds, characterized by reduced respiratory airflow followed by SpO_2 desaturation and an associated increase in heart rate.

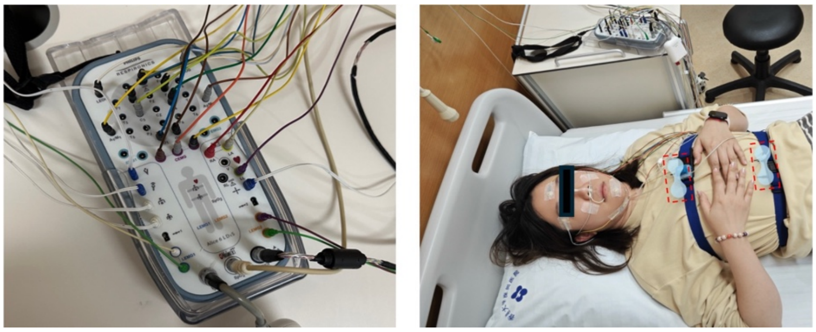

Fig. S24. Simultaneous monitoring setup with HELP and PSG. The illustrated positions of the HELP patches correspond to their actual placement on the skin of the chest and abdomen.

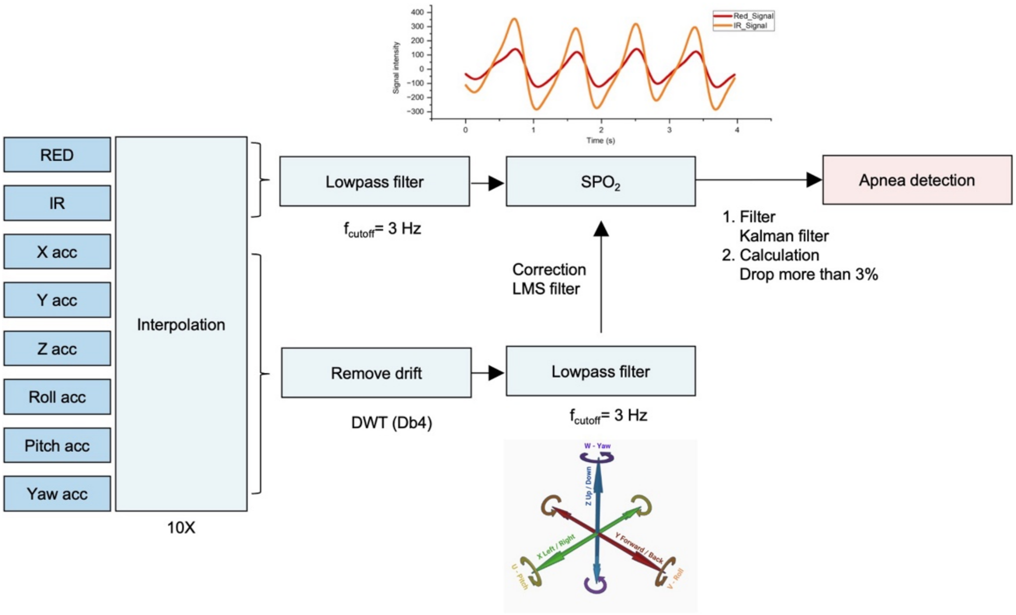

Fig. S25. Signal processing workflow for SpO_2 calculation.

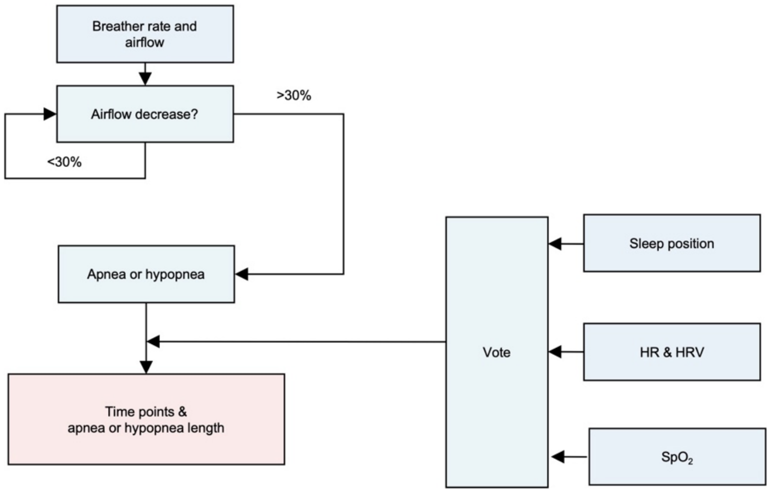

Fig. S26. Algorithmic workflow for sleep apnea-hypopnea event detection. The algorithm begins with preprocessing to extract respiratory rate and airflow variations. A significant reduction in airflow (e.g., >20% decrease from baseline) triggers identification of a potential respiratory event. By integrating multimodal data including sleep position, heart rate, heart rate variability, and blood oxygen saturation (SpO_2), the algorithm classifies event type and outputs the timing, duration, and severity of respiratory depression and apnea episodes.

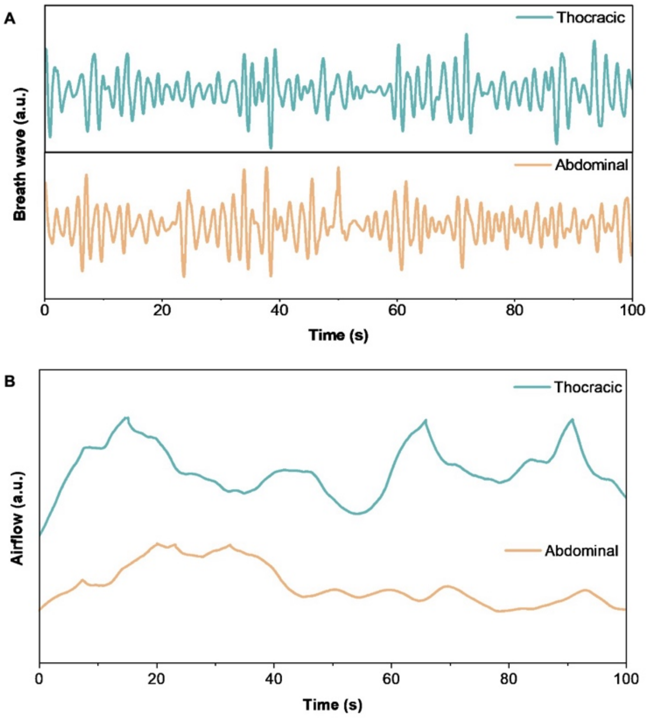

Fig. S27. Thoraco-abdominal breathing synchrony and tidal volume coherence in normal respiration. (**A**) Synchronized respiratory waveforms demonstrating coordinated thoracic and abdominal activity during normal breathing. (**B**) Consistent tidal volume estimation derived from both thoracic and abdominal movements, confirming the coherence of volume displacement between the two compartments during unobstructed respiration.

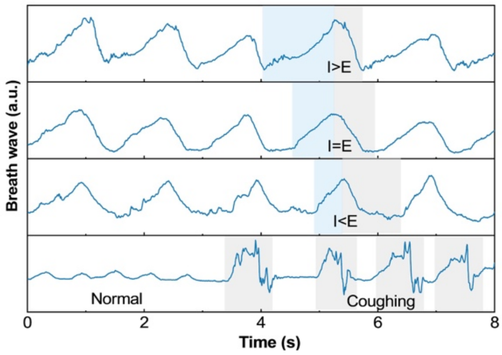

Fig. S28. Recognition of voluntarily controlled breathing patterns with varying inspiratory/expiratory ratios using HELP. I, inhealing time; E, exhealing time.

Movie S1. Robust interfacial integrity of the porous conductive connector demonstrated by mechanical pulling, showing no delamination or LM leakage.

REFERENCES

1. S. D. Shukla, K. Swaroop Vanka, A. Chavelier, M. D. Shastri, M. M. Tambuwala, H. A. Bakshi, K. Pabreja, M. Q. Mahmood, R. F. O'Toole, "Chronic respiratory diseases: An introduction and need for novel drug delivery approaches" in *Targeting Chronic Inflammatory Lung Diseases Using Advanced Drug Delivery Systems* (Elsevier, 2020), pp. 1–31.

2. W. W. Labaki, M. K. Han, Chronic respiratory diseases: A global view. *Lancet Respir. Med.* **8**, 531–533 (2020).

3. G. Viegi, S. Maio, S. Fasola, S. Baldacci, Global burden of chronic respiratory diseases. *J. Aerosol Med. Pulm. Drug Deliv.* **33**, 171–177 (2020).

4. L. Taylor, X. Ding, D. Clifton, H. Lu, Wearable vital signs monitoring for patients with asthma: A review. *IEEE Sensors J.* **23**, 1734–1751 (2020).

5. F.-A. Coutu, O. C. Iorio, B. A. Ross, Remote patient monitoring strategies and wearable technology in chronic obstructive pulmonary disease. *Front. Med.* **10**, 1236598 (2023).

6. B. A. Vicente, R. Sebastião, V. Sencadas, Wearable devices for respiratory monitoring. *Adv. Funct. Mater.* **34**, 2404348 (2024).

7. M. Chu, T. Nguyen, V. Pandey, Y. Zhou, H. N. Pham, R. Bar-Yoseph, S. Radom-Aizik, R. Jain, D. M. Cooper, M. Khine, Respiration rate and volume measurements using wearable strain sensors. *NPJ Digit. Med.* **2**, 8 (2019).

8. R. A. Wise, "Chronic obstructive pulmonary disease (COPD)—Lung and airway disorders" (MSD Manual, 2024). www.msdmanuals.com/home/lung-and-airway-disorders/chronic-obstructive-pulmonary-disease-copd/chronic-obstructive-pulmonary-disease-copd.

9. GBD Chronic Respiratory Disease Collaborators, Prevalence and attributable health burden of chronic respiratory diseases, 1990-2017: A systematic analysis for the Global Burden of Disease Study 2017. *Lancet Respir. Med.* **8**, 585–596 (2020).

10. H. Souri, H. Banerjee, A. Jusufi, N. Radacsi, A. A. Stokes, I. Park, M. Sitti, M. Amjadi, Wearable and stretchable strain sensors: Materials, sensing mechanisms, and applications. *Adv. Intell. Syst.* **2**, 2000039 (2020).

11. B. Laufer, F. Hoeflinger, P. D. Docherty, N. A. Jalal, S. Krueger-Ziolek, S. J. Rupitsch, L. Reindl, K. Moeller, Characterisation and quantification of upper body surface motions for tidal volume determination in lung-healthy individuals. *Sensors* **23**, 1278 (2023).

12. M. S. Rahman, S. Chowdhury, M. Rasheduzzaman, A. B. M. S. U. Doulah, Artificial intelligence-based algorithms and healthcare applications of respiratory inductance plethysmography: A systematic review. *Algorithms* **17**, 261 (2024).

13. D. M. Caretti, P. V. Pullen, L. A. Premo, W. D. Kuhlmann, Reliability of respiratory inductive plethysmography for measuring tidal volume during exercise. *Am. Ind. Hyg. Assoc. J.* **55**, 918–923 (1994).

14. S. Kwon, H. S. Kim, K. Kwon, H. Kim, Y. S. Kim, S. H. Lee, Y.-T. Kwon, J.-W. Jeong, L. M. Trotti, A. Duarte, W.-H. Yeo, At-home wireless sleep monitoring patches for the clinical assessment of sleep quality and sleep apnea. *Sci. Adv.* **9**, eadg9671 (2023).

15. J. Zhong, Z. Li, M. Takakuwa, D. Inoue, D. Hashizume, Z. Jiang, Y. Shi, L. Ou, M. O. G. Nayeem, S. Umezu, K. Fukuda, T. Someya, Smart face mask based on an ultrathin pressure sensor for wireless monitoring of breath conditions. *Adv. Mater.* **34**, e2107758 (2022).

16. L. Ye, F. Wu, R. Xu, D. Zhang, J. Lu, C. Wang, A. Dong, S. Xu, L. Xue, Z. Fan, L. Xu, K. Li, D. Li, A. Kursumovic, R. Zhao, R. Tang, L. Qiu, H. Wang, J. L. MacManus-Driscoll, Q. Jing, W. Li, H. Yang, Face mask integrated with flexible and wearable manganite oxide respiration sensor. *Nano Energy* **112**, 108460 (2023).

17. P. Q. Nguyen, L. R. Soenksen, N. M. Donghia, N. M. Angenent-Mari, H. de Puig, A. Huang, R. Lee, S. Slomovic, T. Galbersanini, G. Lansberry, H. M. Sallum, E. M. Zhao, J. B. Niemi, J. J. Collins, Wearable materials with embedded synthetic biology sensors for biomolecule detection. *Nat. Biotechnol.* **39**, 1366–1374 (2021).

18. S. Gong, X. Zhang, X. A. Nguyen, Q. Shi, F. Lin, S. Chauhan, Z. Ge, W. Cheng, Hierarchically resistive skins as specific and multimetric on-throat wearable biosensors. *Nat. Nanotechnol.* **18**, 889–897 (2023).

19. S. Mirjalali, S. Peng, Z. Fang, C.-H. Wang, S. Wu, Wearable sensors for remote health monitoring: Potential applications for early diagnosis of Covid-19. *Adv. Mater. Technol.* **7**, 2100545 (2022).

20. M. Amjadi, K.-U. Kyung, I. Park, M. Sitti, Stretchable, skin-mountable, and wearable strain sensors and their potential applications: A review. *Adv. Funct. Mater.* **26**, 1678–1698 (2016).

21. Z. Sun, S. Yang, P. Zhao, J. Zhang, Y. Yang, X. Ye, X. Zhao, N. Cui, Y. Tong, Y. Liu, X. Chen, Q. Tang, Skin-like ultrasensitive strain sensor for full-range detection of human health monitoring. *ACS Appl. Mater. Interfaces* **12**, 13287–13295 (2020).

22. S. Aftab, G. Koyyada, M. Mukhtar, F. Kabir, G. Nazir, S. A. Memon, M. Aslam, M. A. Assiri, J. H. Kim, Laser-induced graphene for advanced sensing: Comprehensive review of applications. *ACS Sens.* **9**, 4536–4554 (2024).

23. C. Tang, M. Xu, W. Yi, Z. Zhang, E. Occhipinti, C. Dong, D. Ravenscroft, S.-M. Jung, S. Lee, S. Gao, J. M. Kim, L. G. Occhipinti, Ultrasensitive textile strain sensors redefine wearable silent speech interfaces with high machine learning efficiency. *npj Flex. Electron.* **8**, 27 (2024).

24. Y. Wang, Y. Xie, Interfacial interaction-induced super-wettability of gallium-based liquid metals: A review. *J. Mater. Chem. A* **12**, 7396–7417 (2024).

25. X. Li, E. Rytkin, Q. Zhao, P. Bhat, A. Pfenniger, L. Yin, X. Huang, L. Yang, B. Yang, A. Burrell, A. Mikhailov, R. Arora, I. R. Efimov, H. Zhao, High-resolution liquid metal-based stretchable electronics enabled by colloidal self-assembly and microtransfer printing. *Sci. Adv.* **11**, eadw3044 (2025).

26. D. Y. Choi, M. H. Kim, Y. S. Oh, S.-H. Jung, J. H. Jung, H. J. Sung, H. W. Lee, H. M. Lee, Highly stretchable, hysteresis-free ionic liquid-based strain sensor for precise human motion monitoring. *ACS Appl. Mater. Interfaces* **9**, 1770–1780 (2017).

27. J. Chen, J. Zhang, Z. Luo, J. Zhang, L. Li, Y. Su, X. Gao, Y. Li, W. Tang, C. Cao, Q. Liu, L. Wang, H. Li, Superelastic, sensitive, and low hysteresis flexible strain sensor based on wave-patterned liquid metal for human activity monitoring. *ACS Appl. Mater. Interfaces* **12**, 22200–22211 (2020).

28. Y. Li, A. Veronica, J. Ma, H. Y. Y. Nyein, Materials, structure, and interface of stretchable interconnects for wearable bioelectronics. *Adv. Mater.*, **37**, e2408456 (2025).

29. J. Zhu, J. Li, Y. Tong, T. Hu, Z. Chen, Y. Xiao, S. Zhang, H. Yang, M. Gao, T. Pan, H. Cheng, Y. Lin, Recent progress in multifunctional, reconfigurable, integrated liquid metal-based stretchable sensors and standalone systems. *Prog. Mater. Sci.* **142**, 101228 (2024).

30. M. Kim, H. Lim, S. H. Ko, Liquid metal patterning and unique properties for next-generation soft electronics. *Adv. Sci.* **10**, e2205795 (2023).

31. Q. Zhuang, K. Yao, M. Wu, Z. Lei, F. Chen, J. Li, Q. Mei, Y. Zhou, Q. Huang, X. Zhao, Y. Li, X. Yu, Z. Zheng, Wafer-patterned, permeable, and stretchable liquid metal microelectrodes for implantable bioelectronics with chronic biocompatibility. *Sci. Adv.* **9**, eadg8602 (2023).

32. Y.-H. Wu, R.-M. Zhen, H.-Z. Liu, S.-Q. Liu, Z.-F. Deng, P.-P. Wang, S. Chen, L. Liu, Liquid metal fiber composed of a tubular channel as a high-performance strain sensor. *J. Mater. Chem. C* **5**, 12483–12491 (2017).

33. Y. Park, J. Jung, Y. Lee, D. Lee, J. J. Vlassak, Y.-L. Park, Liquid-metal micro-networks with strain-induced conductivity for soft electronics and robotic skin. *npj Flex. Electron.* **6**, 81 (2022).

34. J. Li, Z. Yao, X. Meng, X. Zhang, Z. Wang, J. Wang, G. Ma, L. Liu, J. Zhang, S. Niu, Z. Han, L. Ren, High-fidelity, low-hysteresis bionic flexible strain sensors for soft machines. *ACS Nano* **18**, 2520–2530 (2024).

35. B. Yao, X. Lü, Y. Wang, N. Bai, C. Chen, S. Wang, H. Su, Y. Zhang, Ultrasensitive, highly stable, and stretchable strain sensor using gated liquid metal channel. *Adv. Funct. Mater.* **34**, 2314298 (2024).

36. B. Xu, M. Yang, W. Cheng, X. Li, X. Xu, W. Li, H. Zhang, M. Zhou, Precision aerosol-jet micropatterning of liquid metal for high-performance flexible strain sensors. *Nat. Commun.* **16**, 7920 (2025).

37. P. A. Lopes, D. F. Fernandes, A. F. Silva, D. G. Marques, A. T. de Almeida, C. Majidi, M. Tavakoli, Bi-phasic Ag-In-Ga-embedded elastomer inks for digitally printed, ultra-stretchable, multi-layer electronics. *ACS Appl. Mater. Interfaces* **13**, 14552–14561 (2021).

38. P. A. Lopes, H. Paisana, A. T. De Almeida, C. Majidi, M. Tavakoli, Hydroprinted electronics: Ultrathin stretchable Ag-in-Ga E-skin for bioelectronics and human-machine interaction. *ACS Appl. Mater. Interfaces* **10**, 38760–38768 (2018).

39. M. Tavakoli, M. H. Malakooti, H. Paisana, Y. Ohm, D. G. Marques, P. Alhais Lopes, A. P. Piedade, A. T. de Almeida, C. Majidi, EGaIn-assisted room-temperature sintering of silver nanoparticles for stretchable, inkjet-printed, thin-film electronics. *Adv. Mater.* **30**, e1801852 (2018).

40. T. Nagasawa, H. Hagiwara, “Workload induces changes in hemodynamics, respiratory rate and heart rate variability,” in *2016 IEEE 16th International Conference on Bioinformatics and Bioengineering (BIBE)* (IEEE, 2016), pp. 176–181.

41. H. Qin, N. Steenbergen, M. Glos, N. Wessel, J. F. Kraemer, F. Vaquerizo-Villar, T. Penzel, The different facets of heart rate variability in obstructive sleep apnea. *Front. Psych.* **12**, 642333 (2021).

42. S. SrinivasVellela, S. P. Praveen, D. Roja, A. R. Krishna, N. Purimetla, T. Rao, K. K. Kumar, “Fusion-infused hypnocare: Unveiling real-time instantaneous heart rates for remote diagnosis of sleep apnea,” in *2024 International Conference on Knowledge Engineering and Communication Systems (ICKECS)* (IEEE, 2024), vol. 4, pp. 1–5.

43. M. Liu, M.-C. Huang, Asthma pattern identification via continuous diaphragm motion monitoring. *IEEE Trans. Multi Scale Comput. Syst.* **1**, 76–84 (2015).

44. R. Boulding, R. Stacey, R. Niven, S. J. Fowler, Dysfunctional breathing: A review of the literature and proposal for classification. *Eur. Respir. Rev.* **25**, 287–294 (2016).

45. J. M. Marin Trigo, M. Marin-Oto, J. P. de Torres, C. Cabrera, I. Solanes, C. Martinez, N. Toledo, G. Peces-Barba, C. Amado, L. Vigil, C. Casanova, Nocturnal hypoxaemia in COPD. Prevalence and clinical characteristics. *Eur. Respir. J.* **50**, PA3640 (2017).

46. J. C. Hogg, Pathophysiology of airflow limitation in chronic obstructive pulmonary disease. *Lancet* **364**, 709–721 (2004).

47. M. Sarkar, R. Bhardwaz, I. Madabhavi, M. Modi, Physical signs in patients with chronic obstructive pulmonary disease. *Lung India* **36**, 38–47 (2019).

48. S. Zhao, D. Liu, F. Yan, Wearable resistive-type stretchable strain sensors: Materials and applications. *Adv. Mater.* **37**, e2413929 (2025).

49. W. Cheng, Z. Luo, C. Wang, T. Zhao, N. Xiang, Soft crawling robot integrated with liquid metal-based flexible strain sensor and closed-loop feedback control. *Sens. Actuators A Phys.* **371**, 115316 (2024).

50. Y. Wu, Y. Zhou, W. Asghar, Y. Liu, F. Li, D. Sun, C. Hu, Z. Wu, J. Shang, Z. Yu, R.-W. Li, H. Yang, Liquid metal-based strain sensor with ultralow detection limit for human–machine interface applications. *Adv. Intell. Syst.* **3**, 2000235 (2021).

51. H. Wu, H. Qi, X. Wang, Y. Qiu, K. Shi, H. Zhang, Z. Zhang, W. Zhang, Y. Tian, Stretchable, sensitive, flexible strain sensor incorporated with patterned liquid metal on hydrogel for human motion monitoring and human–machine interaction. *J. Mater. Chem. C Mater. Opt. Electron. Devices* **10**, 8206–8217 (2022).

52. J. Xu, H. Guo, H. Ding, Q. Wang, Z. Tang, Z. Li, G. Sun, Printable and recyclable conductive ink based on a liquid metal with excellent surface wettability for flexible electronics. *ACS Appl. Mater. Interfaces* **13**, 7443–7452 (2021).

53. Y. Wang, W. Qin, M. Yang, Z. Tian, W. Guo, J. Sun, X. Zhou, B. Fei, B. An, R. Sun, S. Yin, Z. Liu, High linearity, low hysteresis $Ti_3C_2T_x$ MXene/AgNW/liquid metal self-healing strain sensor modulated by dynamic disulfide and hydrogen bonds. *Adv. Funct. Mater.* **33**, 2301587 (2023).

54. G. Li, M. Zhang, S. Liu, M. Yuan, J. Wu, M. Yu, L. Teng, Z. Xu, J. Guo, G. Li, Z. Liu, X. Ma, Three-dimensional flexible electronics using solidified liquid metal with regulated plasticity. *Nat. Electron.* **6**, 154–163 (2023).

55. H. Zhu, Z. Sun, X. Wang, H. Xia, A high-performance strain sensor for the detection of human motion and subtle strain based on liquid metal microwire. *Nanomaterials* **14**, 231 (2024).

56. X. Li, T. Hua, B. Xu, Electromechanical properties of a yarn strain sensor with graphene-sheath/polyurethane-core. *Carbon* **118**, 686–698 (2017).

57. J. Shintake, Y. Piskarev, S. H. Jeong, D. Floreano, Ultrastretchable strain sensors using carbon black-filled elastomer composites and comparison of capacitive versus resistive sensors. *Adv. Mater. Technol.* **3**, 1700284 (2018).

58. Q. Xia, S. Wang, W. Zhai, C. Shao, L. Xu, D. Yan, N. Yang, K. Dai, C. Liu, C. Shen, Highly linear and low hysteresis porous strain sensor for wearable electronic skins. *Compos. Commun.* **26**, 100809 (2021).

59. B. Liu, B. Lan, L. Shi, Y. Cheng, J. Sun, R. Wang, Batch fabrication of flexible strain sensors with high linearity and low hysteresis for health monitoring and motion detection. *ACS Appl. Mater. Interfaces* **16**, 36821–36831 (2024).

60. W. Zhu, X. Mo, Z. Wang, H. Liu, J. Chen, L. Wang, D. Shou, Machine learning-enhanced low-hysteresis conductive auxetic strain sensors with curved re-entrant honeycomb structures based on MXene/graphene for human rehabilitation training. *Chem. Eng. J.* **505**, 159539 (2025).

61. L. Zheng, D. Liu, J. Dai, Z. Hao, W. Wu, X. Yin, Flexible antibacterial strain sensor with low electrical hysteresis, ultralow detection limit, and wide linear sensing range for human motion monitoring and human–machine interaction. *Chem. Eng. J.* **500**, 157289 (2024).

62. H.-J. Kim, A. Thukral, C. Yu, Highly sensitive and very stretchable strain sensor based on a rubbery semiconductor. *ACS Appl. Mater. Interfaces* **10**, 5000–5006 (2018).

63. J. Zou, X. Jing, Z. Chen, S.-J. Wang, X.-S. Hu, P.-Y. Feng, Y.-J. Liu, Multifunctional organohydrogel with ultralow-hysteresis, ultrafast-response, and whole-strain-range linearity for self-powered sensors. *Adv. Funct. Mater.* **33**, 2213895 (2023).

64. C. Ye, T. Cang, J. Zhu, Z. Wang, X. Li, Soft thermoplastic polyurethane/silver nanowire membranes with low hysteresis for large strain sensing and joule heating. *ACS Appl. Polym. Mater.* **6**, 11149–11159 (2024).

65. Z. Shen, Z. Zhang, N. Zhang, J. Li, P. Zhou, F. Hu, Y. Rong, B. Lu, G. Gu, High-stretchability, ultralow-hysteresis conducting polymer hydrogel strain sensors for soft machines. *Adv. Mater.* **34**, e2203650 (2022).

66. W. Huang, X. Wang, F. Luo, X. Zhao, K. Chen, Y. Qin, Ultrastretchable, ultralow hysteresis, high-toughness hydrogel strain sensor for pressure recognition with deep learning. *ACS Appl. Mater. Interfaces* **16**, 49834–49844 (2024).

67. B. Wang, M. Liu, H. M. Wang, S. Yang, X. Li, S. Yin, Z. Xu, Q. Liu, Y. Lu, L. Yang, M. Li, Dual-strain adaptive conductive channels conferred sensing rope with ultrahigh linearity, wide working range, and yoga-Asana-monitoring capability. *ACS Sens.* **10**, 6095–6105 (2025).

68. H. Hertz, The contact of elastic solids. *J. Reine Angew. Math.* **92**, 156–171 (1881).

69. E. Winkler, *Die Lehre von der Elasticität und Festigkeit* (Dominicus, 1867).
